# Short term evaluation of respiratory effort by premature infants supported with bubble nasal continuous airway pressure using Seattle-PAP and a standard bubble device

**DOI:** 10.1371/journal.pone.0193807

**Published:** 2018-03-28

**Authors:** Stephen E. Welty, Craig G. Rusin, Larissa I. Stanberry, George T. Mandy, Alfred L. Gest, Jeremy M. Ford, Carl H. Backes, C. Peter Richardson, Christopher R. Howard, Thomas N. Hansen, Charles V. Smith

**Affiliations:** 1 Department of Pediatrics, The University of Washington College of Medicine, Seattle, Washington, United States of America; 2 Department of Pediatrics, Baylor College of Medicine, Houston, Texas, United States of America; 3 Center for Developmental Therapeutics, Department of Pediatrics, Seattle Children’s Research Institute, Seattle, Washington, United States of America; 4 Department of Pediatrics West Virginia University School of Medicine, Morgantown, West Virginia, United States of America; 5 Department of Pediatrics and Center for Perinatal Research, the Ohio State University College of Medicine and School of Public Health, Columbus, Ohio, United States of America; TNO, NETHERLANDS

## Abstract

**Background:**

Almost one million prematurely born infants die annually from respiratory insufficiency, predominantly in countries with limited access to respiratory support for neonates. The primary hypothesis tested in the present study was that a modified device for bubble nasal continuous positive airway pressure (Bn-CPAP) would provide lower work of spontaneous breathing, estimated by esophageal pressure-rate products.

**Methods:**

Infants born <32 weeks gestation and stable on Bn-CPAP with FiO_2_ <0.30 were studied within 72 h following delivery. Esophageal pressures during spontaneous breathing were measured during 2 h on standard Bn-CPAP, then 2 h with Bn-CPAP using a modified bubble device presently termed Seattle-PAP, which produces a different pattern of pressure fluctuations and which provided greater respiratory support in preclinical studies, then 2 h on standard Bn-CPAP.

**Results:**

All 40 infants enrolled completed the study and follow-up through 36 wks post menstrual age or hospital discharge, whichever came first. No infants were on supplemental oxygen at completion of follow-up. No infants developed pneumothoraces or nasal trauma, and no adverse events attributed to the study were observed. Pressure-rate products on the two devices were not different, but effort of breathing, assessed by areas under esophageal pressure-time curves, was lower with Seattle-PAP than with standard Bn-CPAP.

**Conclusion:**

Use of Seattle-PAP to implement Bn-CPAP lowers the effort of breathing exerted even by relatively healthy spontaneously breathing premature neonates. Whether the lower effort of breathing observed with Seattle-PAP translates to improvements in neonatal mortality or morbidity will need to be determined by studies in appropriate patient populations.

## Introduction

Worldwide, roughly one million premature infants die each year with respiratory distress syndrome [1]. Because of advances in neonatal medicine, such as routine use of antenatal corticosteroids, mechanical ventilation, and pulmonary surfactant replacement therapy, few of these deaths occur in developed countries, where the primary lung burden is chronic lung disease of prematurity, commonly termed bronchopulmonary dysplasia (BPD) [2, 3]. Unfortunately, because of technical and economic barriers, the use of these life-saving therapies is limited in low and middle income countries (LMICs) [4–6]. In developed countries, the standard of care for respiratory support in neonates with respiratory failure is evolving toward the use of nasal continuous positive airway pressure (n-CPAP), mainly because of the association with lower rates of BPD than are observed with more invasive modes of support [7, 8].

Evidence that even brief exposures to mechanical ventilation may cause lung injury has resulted in a growing number of nurseries in high, middle, and low income countries migrating to nasal CPAP (n-CPAP) as the initial treatment for preterm infants with RDS [7, 8]. Intubation, administration of exogenous surfactant, and mechanical ventilation are then used as rescue therapies when necessary and if available. Two meta-analyses [8, 9] have also found that n-CPAP at birth produces lower rates of BPD or the combined outcome of BPD or death. The Committee on the Fetus and Newborn of the American Academy of Pediatrics (AAP) summarized the current trials comparing n-CPAP at birth and selective surfactant administration with routine intubation and prophylactic surfactant treatment and found lower rates of the combined outcomes of death and BPD in infants receiving n-CPAP as the initial therapy [7]. Unfortunately, substantial and highly variable numbers (18–67%) of infants treated initially with n-CPAP develop respiratory failure and require intubation, surfactant administration, and mechanical ventilation, with the attendant complications; in LMICs and other resource-limited health care institutions, many of these infants die [2, 10].

More recently, bubble nasal-CPAP (Bn-CPAP) has re-emerged as a strategy to address the high failure rates associated with conventional (usually ventilator-derived) CPAP [10, 11]. Pillow reported that preterm lambs with RDS treated with Bn-CPAP had better ventilation, oxygenation, and lung volume recruitment than did lambs treated with ventilator-derived CPAP [12]. In 18 preterm infants with mild RDS, Courtney found no differences in lung mechanics, but the infants on Bn-CPAP had higher TcPO_2_ levels than did the infants on Vn-CPAP [13]. Courtney’s study obtained measurements after only 5 min of stabilization, which may have been too short for the lungs to adapt completely to each new setting, thereby underestimating possible impacts of changes in support by Bn-CPAP.

In a randomized controlled trial of preterm infants with RDS, Bahman-Bijari compared outcomes between 25 infants born weighing 1000 to 2000 g treated with Bn-CPAP from birth with another 25 preterm infants treated with CPAP delivered by a mechanical ventilator (Vn-CPAP) [14]. The infants treated with Bn-CPAP had higher 72 h survival rates than did the infants treated with Vn-CPAP (100 versus 59%, different by log rank), lower rates of CPAP failure (4% versus 28%, p = 0.02), and lower costs of hospitalization ($947.3±726 versus $1436.7±934, respectively, (*P* = 0.04).

Tapia randomized 250 preterm infants to receive Bn-CPAP with the INSURE protocol with infants treated with oxygen and, if required, mechanical ventilation and rescue surfactant (Oxygen/MV group) [15]. Of the infants treated with Bn-CPAP, 29.8% required mechanical ventilation, versus 46.4% of the infants in the Oxygen/MV group (p = 0.001). Similarly, 27.5% of the infants in the Bn-CPAP infants were given surfactant, versus 46.4% of the infants in the Oxygen/MV infants (p = 0.002). Tagare studied 114 preterm infants with RDS randomized to treatment with Vn-CPAP or Bn-CPAP, with an end point of CPAP failure [16]. More infants in the Bn-CPAP group were treated successfully than were those in the Vn-CPAP group (82.5% versus 63.2%, p = 0.03). Nasal trauma was observed more commonly in the Bn-CPAP infants than in the Vn-CPAP infants (12 versus 4, p = 0.03), but short-term morbidity and mortality were similar in the two groups. Martin reviewed the randomized controlled trials, along with a number of observational studies, and reported lower rates of CPAP failure with Bn-CPAP in LMICs [11].

Based on the current evidence, infants treated with Bn-CPAP have slightly better outcomes than do infants treated with Vn-CPAP. Bn-CPAP is inexpensive and relatively easy to use, which makes this mode of support even more appropriate for use in LMICs. However, Bn-CPAP in developed countries is insufficient to prevent respiratory failure in about 24% of infants born weighing <1250 g at birth and 50% of infants born weighing <750 g [17]. In resourced health care institutions, failure of support by Bn-CPAP results in endotracheal intubation and invasive mechanical ventilation (MV), often with administration of exogenous pulmonary surfactant [2, 17, 18]. In resource-limited settings, failure of Bn-CPAP in many cases would result in death of the infant.

We developed a device for use with Bn-CPAP that we termed Seattle-PAP, which [19]in preclinical studies with juvenile rabbits lavaged to create substantial pulmonary deficiency, provided greater levels of respiratory support than did conventional Bn-CPAP [20, 21]. Decreasing the effort required to breathe by infants on Bn-CPAP should decrease the numbers of infants who fail noninvasive respiratory support due to apnea precipitated by respiratory muscle fatigue, over a wide range of acutely impaired lung function.

The purpose of the present trial was to compare Bn-CPAP produced with Seattle-PAP with traditional Bn-CPAP in prematurely born neonates. The primary goal was to test the working hypothesis that work of breathing, as estimated by esophageal pressure-rate products (PRP), in stable preterm neonates receiving respiratory support on Bn-CPAP would be lower on Seattle-Bn-CPAP than on Bn-CPAP provided by the present standard with the Fisher & Paykel device (FP-Bn-CPAP). Because other studies, particularly in humans, have indicated that respiratory fatigue is more closely associated with less simplified measures of effort of breathing [22–24], esophageal pressure-time curves also were assessed as indices of effort of breathing.

## Materials and methods

### Study infants

The inclusion criteria for this study were infants born at ≤32 weeks of gestational age and admitted to the Pavilion for Women NICU at Texas Children’s Hospital (TCH), within the first 72 h of life, and stable on standard Bn-CPAP, defined as maintaining arterial saturations (S_a_O_2_) within the targeted range of 90–95%, at mean airway pressures of ≤8 cmH_2_O and FiO_2_ ≤0.30. The standard of care at TCH includes the use of the Bn-CPAP device from Fisher & Paykel. Patients with major congenital anomalies or suspected chromosomal abnormalities were excluded from the study. Potential study patients were identified by the PI scanning lists for new admissions who may have met inclusion criteria, after which the patients’ charts were reviewed to determine eligibility using the inclusion/exclusion criteria check list. The families of patients determined to be eligible were approached to describe the study in detail. The family members were given time to review the consent form, ask questions, and decide whether to consent to the study. If the family consented, study personnel were alerted, and the study was undertaken. Patients recruited over a weekend were studied on the following Monday. Recruitment and follow-up occurred during the period from August 5, 2014 through November 5, 2015. The study protocol is registered with ClinTrials.gov (NCT02210026) and was approved by the Baylor College of Medicine (BCM) (H-29620) and Seattle Children’s Hospital (SCH IRB 15203) institutional review boards. Informed consent was obtained for each neonate studied.

We have previously published data on studies comparing Bn-CPAP with Seattle-PAP using 12 lavaged, sedated juvenile rabbits weighing around 1000 g [20]. In these studies, work of breathing was estimated using pressure x rate products (PRPs); the difference in the means for PRPs was 148.24 cmH_2_O/min. The standard deviation of the differences, in the paired comparisons of Bn-CPAP vs. Seattle-PAP, was 154.23 cmH_2_O/min. From these data, for two-tailed alpha of 0.05, sample size calculations, using paired t-tests for repeated observations in the same subject, with Primer of Biostatistics Version 7.0, indicated n = 11 and 14, for powers of 0.80 and 0.90, respectively. To allow for anticipated greater heterogeneity of the human infant population that we needed to characterize and the fact that the infants to be studied would not be sedated, we proposed to study 40 infants, with ongoing review of accumulating data by a data safety monitoring board after the first 10 infants.

### Data collection

Demographic data and device settings were collected manually and included gestational age at birth, gender, birth weight, race, ethnicity, postnatal age at the start of the study and medications administered after delivery and prior to the study. Bn-CPAP device settings were also collected manually and included fraction of inspired oxygen (FiO_2_), CPAP tube depth, and CPAP bias gas flow rate.

Continuous physiologic data were collected and recorded while infants were managed for two h on FP-Bn-CPAP, followed by two h on Seattle-Bn-CPAP, then two h on FP-Bn-CPAP. Additional data obtained included transcutaneous PCO_2_, S_a_O_2_ by pulse oximetry, respiratory rates and esophageal and airway pressure measurements. Physiologic data were automatically time-synchronized and recorded from the individual measurement devices.

Heart rates were collected at 0.5 Hz, and 3 lead electrocardiogram at 240 Hz (GE Solar 8000, Milwaukee, WI), as well as esophageal and airway pressure measurements recorded at 240 Hz (DARCI, SCRI, Seattle, WA). Physiologic data were automatically time-synchronized and recorded from the individual measurement devices by the Sickbay^™^ Platform (Medical Informatics Corp, Houston, TX). Esophageal pressure changes (ΔP_es_) were used to assess changes in pleural pressures. To measure esophageal pressure (P_es_) changes during respiratory cycles, single-lumen 6 Fr (2 mm) esophageal catheters were placed in the distal esophagus, and the pressures were evaluated in near real time to assure proper placement [25]. Airway pressures were measured directly at the nasal interface.

### Signal pre-processing

The infants were not sedated for purposes of this study. The esophageal pressure data were processed prior to analyses, to identify periods of quiet breathing and to remove or correct for cardiac artifacts, infant movements, and other interfering effects. Quiet breathing was defined as groups of at least 10 consistent breaths. From cardiac-filtered P_es_ signals, ΔP_es_ during quiet breathing were calculated as the differences in pressure levels at peak and trough. The time between breaths (Δt) was computed as the difference in time between consecutive peaks. The preprocessing methods resulted in the present calculations being based upon considerably fewer than the 105 segments of one min each remaining after omission of the first 15 min of each epoch for neonate responses to diaper changes and other patient care interactions.

After the pre-processing, the first 15 min of P_es_ signals in each neonate’s two hour epoch were omitted from analyses, by design, as these periods were allotted for patient care activities that we anticipated would interfere with quiet breathing. The remaining P_es_ signal data were split into consecutive one min intervals, and only intervals of quiet breathing containing ten or more quiet breaths were retained for analyses. For each one min interval, ΔP_es_, Δt, and PRP were calculated as averages over the corresponding values for all quiet breaths in that interval; the respiratory rate (RR) of quiet breathing was calculated as a reciprocal of Δt. These one min summaries represent study data used for analyses.

To estimate area under the curve (AUC) per breath, the P_es_-time curve for each breath was smoothed, and the area above the curve was calculated using trapezoidal approximation. The resulting variable was termed AUC as a more familiar convention. AUCs per breath were computed as averages of AUCs over all quiet breaths within each one min interval that contained at least 10 consistent breaths manifesting no swallowing efforts and no body or arm movements. The effort expended per min in breathing by a neonate was approximated as a product of the average AUC per breath and the corresponding RR in each one min interval.

The covariates in the analyses included those thought to influence pulmonary function (gestational age, age at the start of the experiment, and previous surfactant administration), as well as those that were found to be associated with differences in the response variable during exploratory data analyses. Administration of surfactant and antibiotics were included as binary variables that were set to one for the medication being administered and zero if not.

### Statistical methods

Unless specified otherwise, data are presented as mean±standard deviation (SD). Residual analyses were performed using graphical and summary statistics. For the first level analysis, data for each epoch were summarized on a subject level, using mean values for RR and median values for PRP, ΔP_es_, AUC per breath, and AUC per min. These derived summary measures were used for comparisons across the epochs by repeated measures ANOVA and by nonparametric Friedman’s tests. PRP, ΔP_es_, AUC per breath, and AUC per min were not distributed normally, so the data were log transformed for analysis by ANOVA. Pearson’s correlations were used to examine relationships between initial gas flow rates and gestational ages at birth and birth weights.

Longitudinal data derived for each response variable were analyzed additionally using generalized estimating equations (GEE). The results are reported as coefficient estimates ± empirical estimates of their corresponding standard errors. The numbers of data points per epoch used in the analyses were compared on log scale, by repeated measures ANOVA. Signal processing and data analyses were performed with Primer for Biostatistics, version 7.0 [26], in Matlab (R2015a, Mathworks Inc.), and with R [27].

For the first level analysis, data for each epoch were summarized on a subject level using mean values for RR and median values for PRP, ΔP_es_, AUC per breath, and AUC per min. The distributions of PRP, ΔP_es_, AUC per breath, and AUC per min were skewed, so the data were analyzed on log scale using repeated measures ANOVA and on original scale by nonparametric Friedman’s tests.

For the GEE analyses, the null hypothesis for the individual model coefficients was tested using the Wald test statistic [28]. The mean responses for Epochs 1 and 3 were compared using the Wald statistic based on a quadratic form of the differences and the empirical covariance structure. Spectral analysis of the residuals indicated the presence of non-negligible autocorrelations. Using the Quasi Information Criterion (QIC), the first-order autoregressive working correlation matrix was selected for the GEE over the independence structure [29].

The PRP, ΔP_es_, AUC per breath, and AUC per min distributions were right-skewed, and the variables were log transformed for analysis. The model coefficients, β, are interpreted as 100(e^β^ -1) percent changes in response variables for a unit/level change in the covariates. The RR distribution was symmetrical and approximately normal, so the data were analyzed without transformation. The mean heart rates and the overall mean respiratory rates across epochs were analyzed on the original scale using linear mixed effect models.

## Results

All 40 neonates enrolled completed the six h study and follow-up through 36 wks post menstrual age or hospital discharge, whichever came first. No infants were on supplemental oxygen at completion of the study at 36 wks or at discharge from the hospital. No infants developed pneumothoraces or nasal trauma, and no adverse events attributed to the study were observed.

**Fig 1:**
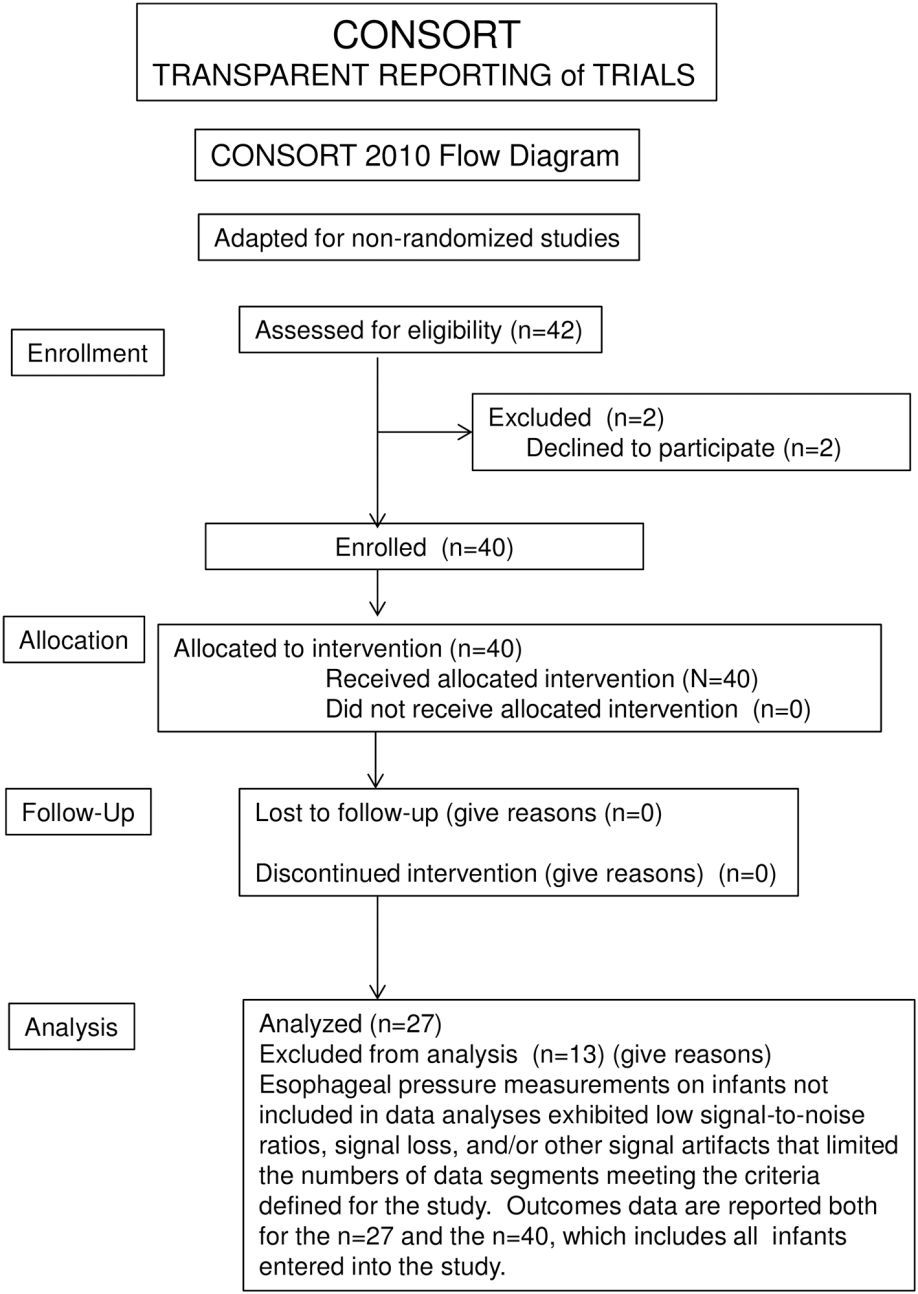
CONSORT flow chart for enrollment and study of infants. Due to the repeated measures design of the present study, enrollment of consecutive infants was neither intended nor attempted. Of the 42 sets of parents approached for enrollment, 40 agreed, and all of these infants completed the study, including follow-up to 36 weeks or discharge from hospital. Thus, follow-up and other data are comprehensive for all 40 of these infants. However, technical issues, many arising from the physical activities, such as body motion, swallowing, and similar actions of the unsedated infants interfering with efforts to record P_es_, limited acquisition of interpretable data in 13 of the 40 infants.

Esophageal pressure measurements acquired on infants 1, 2, 4–7, 9, 13, 17, 22, 27, and 28 exhibited low signal-to-noise ratios, signal loss, and/or other signal artifacts that limited the numbers of data segments meeting the criteria defined for the study. Data from these infants were not included in the analyses. Infant 29 had an episode of airway obstruction during the study, and data from this infant were excluded from analysis (Fig 1). Demographic data for the complete set of 40 enrolled patients, the 27 patients included in data analyses, and the 13 patients whose data were not included were virtually identical, with the exception that greater numbers of breaths were analyzed in the infants whose data were used than for infants for whom data did not meet use criteria (Table 1).

**Table 1:** Demographic and clinical summary statistics for patients in the study.

Variable^1^	Used in the analyses, n = 27	Not used in the analyses, n = 13	p-value
**Gestational age, wks**	29.6±1.7 (29.9)	29.1±1.9 (28.7)	0.36
**Birth weight, g**	1335±245 (1361)	1221±308 (1265)	0.21
**Female**	18 (67)	8(62)	1.0
**Race**			0.81
**Asian**	1 (3)	0(0)	
**Black**	8 (30)	3(23)	
**White**	18 (67)	10(77)	
**Ethnicity**			0.73
**Hispanic**	7 (26)	2 (15)	
**Ampicillin and Gentamicin**	18 (67)	9 (69)	1.0
**Surfactant**	18 (67)	6 (46)	0.30
**Age at start of study, h**	44±13 (47)	47±10 (43)	0.64
**Numbers of quiet breaths**^2^			0.001
**Epoch 1**	912 [529–1677]	422 [141–569]*	
**Epoch 2**	627 [384–1069]	69 [34–468]*	
**Epoch 3**	994 [696–1457]	146 [96–309]*	

^1^Baseline data are summarized as mean±standard deviation (median) for continuous variables and count (%) for categorical variables, respectively.

^2^Breaths in the first 15 min of each epoch are excluded; data on numbers of breaths per epoch exhibit skewed distributions and are presented as median [interquartile range (IQR)].

*Summaries in the not used group exclude data for the first seven patients, for whom data acquisition was compromised. The data were compared using unpaired t-tests for continuous variables (log transformed) and Fisher’s exact test for categorical variables.

Of the 27 neonates for whom the data were analyzed, 12 were administered both surfactant and antibiotics, six received surfactant but did not receive antibiotics, six received antibiotics but no surfactant, and three infants received neither surfactant nor antibiotics. Gestational ages at birth, postnatal ages at start of the study, and birth weights for those who received surfactant or antibiotics were not different in infants thus treated from those who did not receive these therapies (S1 Table). Close to half (21/40) of the neonates completed the study follow-up through 36 wks post menstrual age, whereas the rest of the neonates completed follow-up by discharge from the hospital. TcPCO_2_ measurements were problematic, with considerable within patient variability and missing data. In the 15 patients with complete TcPCO_2_ data, no differences were indicated among epochs 1, 2, and 3, (52.1±1.99, 51.7±2.01 and 52.4 ±2.01 mmHg, respectively).

Differences in bias gas flow rates, 6, 7, or 8 l/min, and tube depths, 5, 6, 7, or 8 cm, (S2 Table) were largely attributable to initial conditions set by the clinical care team. FiO_2_ levels were adjusted to maintain target oxygen saturations between 90 and 95%, which is the standard of care in the TCH nursery. Most neonates studied were receiving room air during most of their 6 h study period. Initial Bn-CPAP tube depth settings did not correlate with gestational age at birth or with birth weights. By linear regression analyses, initial gas flow rates did correlate with birth weights (r = 0.318, P = 0.046), but flow rates did not correlate with gestational ages at birth (r = 0.032, P = 0.843). Following the initial settings, only two neonates had changes in tube depth, both occurring during their respective Epoch 1. Gas flow rates were changed only in one infant, during that neonate’s Epoch 2, and seven neonates had their FiO_2_ levels changed during the 6 h study period.

**Fig 2:**
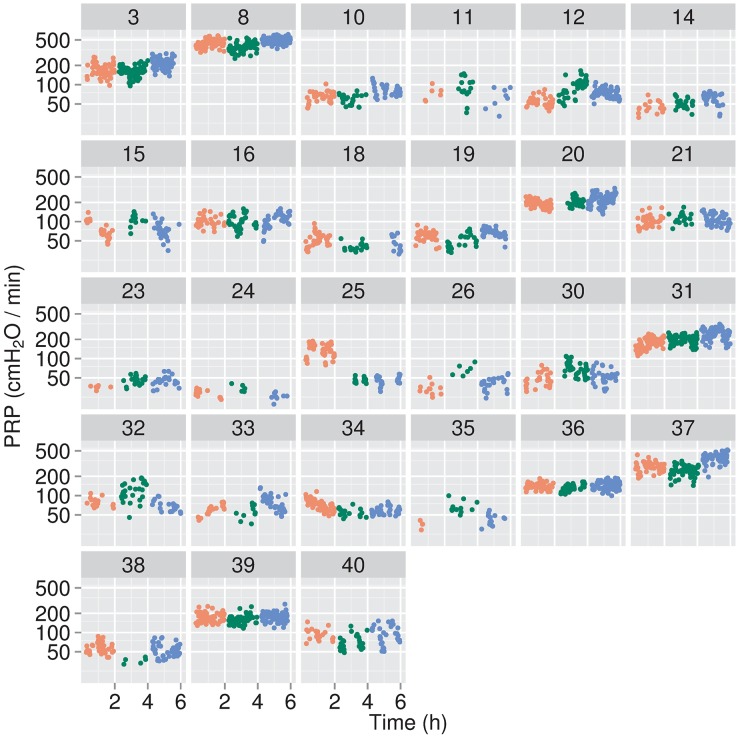
Pressure-rate products (PRP) for individual infants. PRPs were calculated as ΔP_es_ × RR, where ΔP_es_ are the changes in the esophageal pressures between peak exhalation and peak inhalation, corrected to remove cardiac and other artifacts. RRs are the respiratory rates, calculated from the time intervals between quiet breaths. For data analyses, data from the first 15 min of each epoch were omitted, to allow for patient care interactions. The 105 min data collection periods for each epoch were segmented into consecutive one min intervals, and only those intervals containing 10 or more quiet breaths were retained for analysis. The resulting data are means for those respective one min periods and are presented as individual data points for each infant, with red for epoch 1, green for epoch 2, and blue for epoch 3.

**Fig 3:**
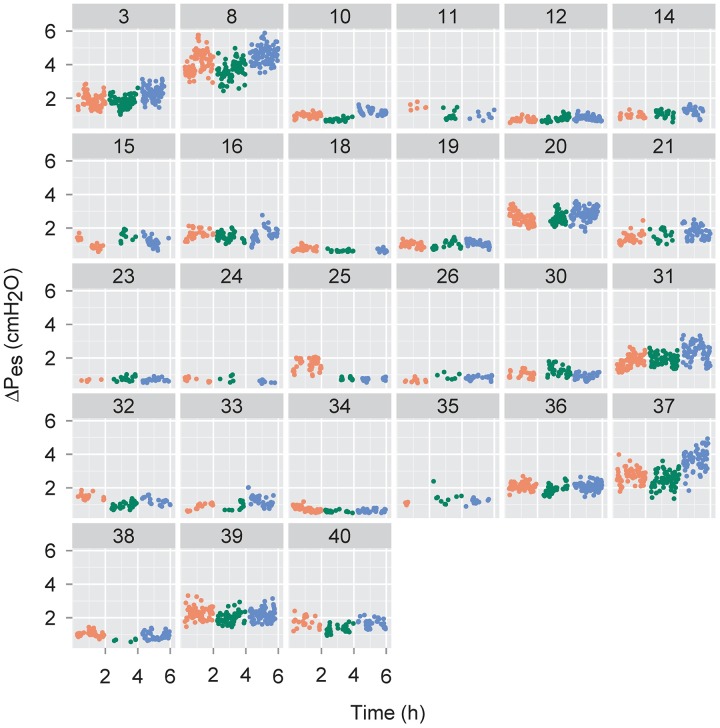
Esophageal pressure changes (ΔP_es_) for individual infants. The ΔP_es_ values measured for each infant in the 1 min intervals in which at least 10 quiet breaths were observed were averaged and are expressed as individual values for that minute. The resulting data are means for those respective one min periods and are presented as individual data points for each infant, as described for Fig 2.

**Fig 4:**
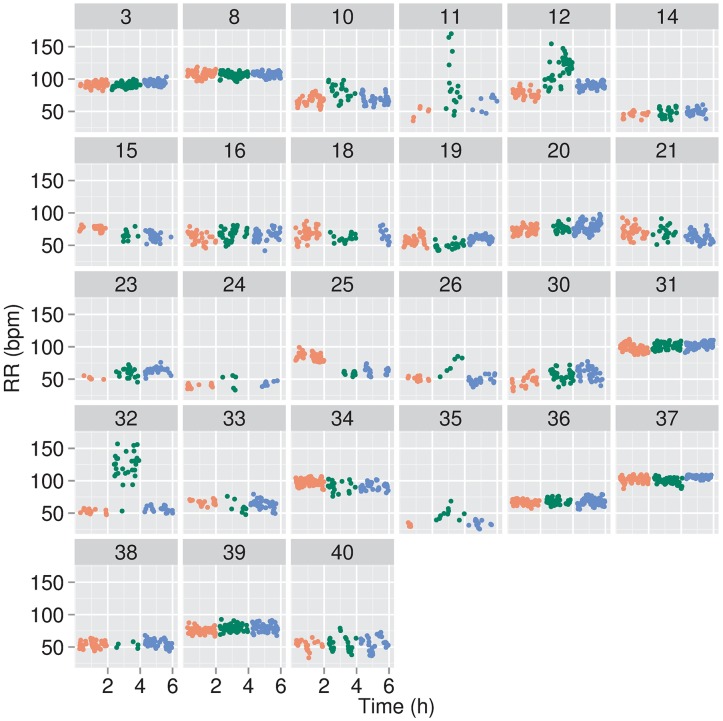
Respiratory rates (RR) for individual infants. RRs were determined and are expressed similarly as for the ΔP_es_ data presented in Fig 3.

**Fig 5:**
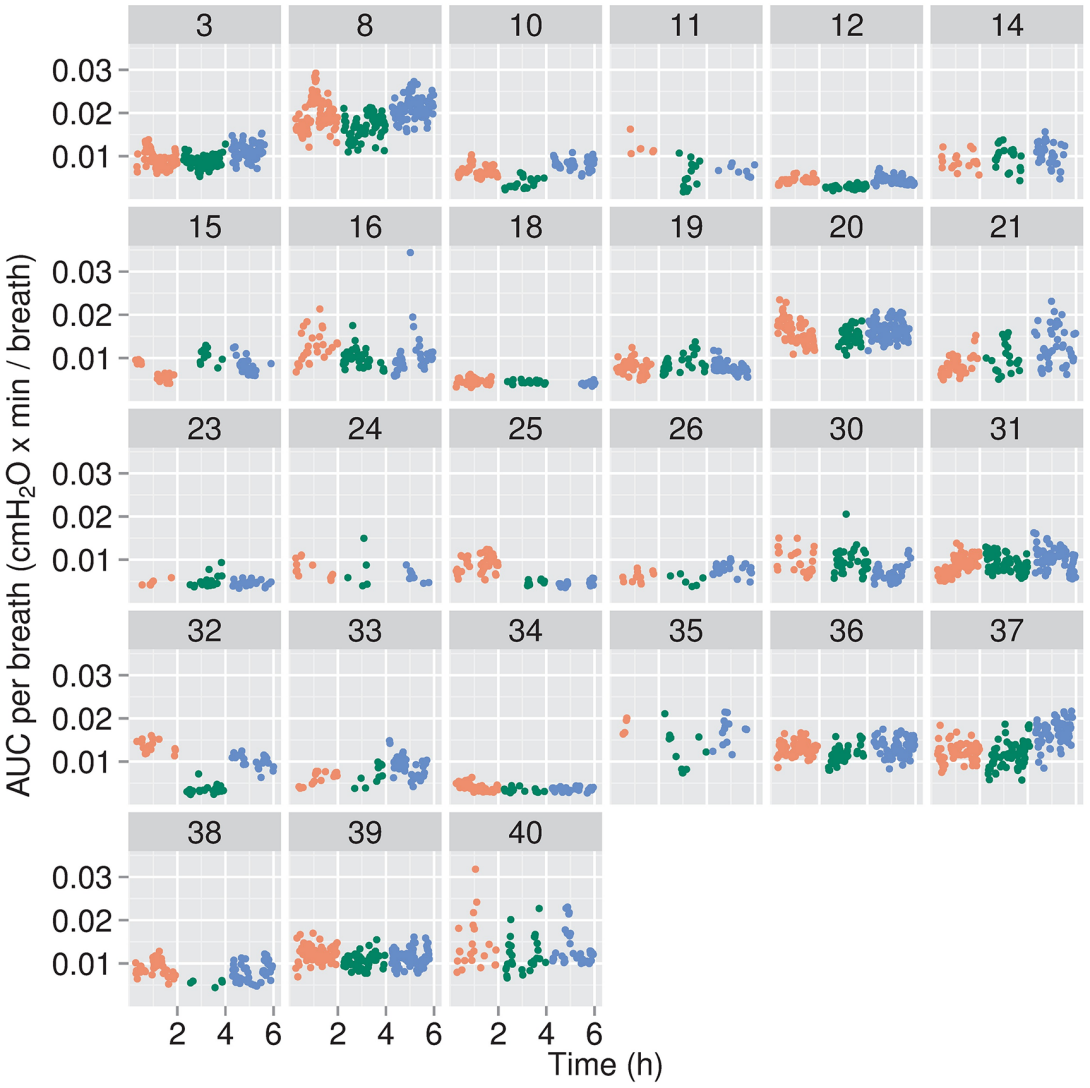
Areas under the P_es_-time curve (AUC) per breath for individual infants. AUCs, more specifically the areas above the ΔP_es_-time curves, were calculated for quiet breaths in one min segments in which at least 10 quiet breaths were observed and are averaged across all quiet breaths in that min interval; the data are presented as cmH_2_O•min.

**Fig 6:**
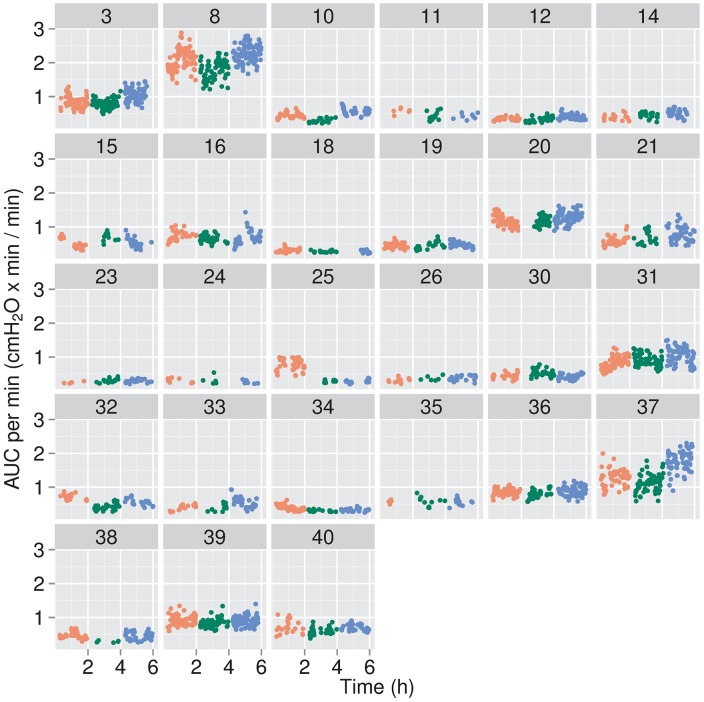
Areas under the curve (AUC) per min for individual infants. AUCs were calculated for quiet breaths in one min segments in which at least 10 quiet breaths were observed and normalized per min, using the RR derived from quiet breaths in that interval for the infant, with the data thus expressed as cmH_2_O•min/min.

Neonate 8 was on FiO_2_ ≤0.30 at the time the investigator signed the checklist to start the study, but early in epoch 1, the FiO_2_ was changed to 0.33. By 24 min into the study, the FiO_2_ was returned to 0.30, with a further decrease to 0.28 for Epochs 2 and 3 (S2 Table). A protocol variance report was recorded on this patient. A second patient, 26, was treated with supplemental oxygen (FiO_2_ of 0.96) for six min in response to a desaturation caused by water condensate flowing into his nasal prongs. This infant returned to breathing room air without any further events. In the 27 patients included in our data analyses, measurements of PRP, ΔP_es_, RR, AUC per breath, and AUC per min exhibited notable interpatient differences, as well as variations within epochs for each patient (Figs 2–6).

The PRPs observed in the infants studied did not differ in the respective epochs (Table 2). Although differences in ΔP_es_ and RR were not indicated in assessment of the data by repeated measures ANOVA, differences (P<0.05) among epochs in these parameters were indicated by nonparametric Friedman tests. AUCs of (P_es_×t) per breath and per min were different among epochs (Table 2), indicating 11 to 16% lower efforts of breathing during respiratory support with Seattle-PAP than during support with conventional Bn-CPAP, even in this relatively healthy group of infants. These findings were consistent with the GEE analyses of longitudinal data (Table 3).

**Table 2:** Inter-Epoch comparisons of effort of breathing.

Response Variable	PRP	ΔP_es_	RR	AUC per Breath	AUC per Min
Units	cmH_2_O/min	cmH_2_O/breath	breaths/min	(cmH_2_O×min)×10^3^	cmH_2_O×min/min
Epoch 1	105.7± 90.0	1.44±0.79	67.1±19.9	9.58±4.01	0.65±0.38
Epoch 2	104.5± 79.4	1.33±0.73	73.8±22.0	8.10±3.75	0.58±0.35
Epoch 3	118.1±111.7	1.53±0.98	68.6±19.0	9.70±4.49	0.69±0.47
					
ANOVA (F, P)	0.54, 0.586	2.50, 0.092	3.01, 0.058	5.93, 0.005	3.50, 0.038
Friedman (χ^2^, P)	4.67, 0.097	7.41, 0.025	6.74, 0.034	12.52, 0.002	7.19, 0.028

The response variables are presented as means±SD computed from the median values for each epoch for each patient, except for RR, for which means were calculated from mean RR per patient per epoch. To calculate AUCs, the P_es_-time curve for each quiet breath was smoothed, and the area above the curve was calculated using trapezoidal approximation. The resulting variable is termed AUC as a more familiar convention. The average AUCs per breath were calculated for the QBs within each one min interval that contained at least 10 QBs. The effort expended per min of breathing is approximated as the product of the average AUC per breath × RR in each one min interval thus calculated. Data among epochs were compared using repeated measures ANOVA, Fisher Exact Test, and Friedman χ^2^, with calculated P values, as indicated.

**Table 3:** GEE model fit of PRP, ΔP_es_, RR, and AUCs, on time and epoch.

Response:	PRP (cmH_2_O/min)	ΔP_es_ (cmH_2_O/breath)	RR (breaths/min)	AUC per Breath (cmH_2_O•min)	AUC per min (cmH_2_O•min/min)
Factor	Estimate±SE (Wald, P)	Estimate±SE (Wald, P)	Estimate±SE (Wald, P)	Estimate±SE (Wald, P)	Estimate±SE (Wald, P)
Time (min)	(5.7±4.1)×10^−4^ (1.88, 0.170)	(5.4±3.7)×10^−4^ (2.22, 0.136)	0.01±0.02 (0.37, 0.545)	(-3.1±4.8)×10^4^ (0.41, 0.520)	(5.8±4.2)×10^−4^ (1.90, 0.168)
Epoch 1	0.04±0.06 (0.40, 0.528)	0.13±0.06 (5.16, 0.023)	-3.81±3. 34 (1.30, 0.254)	0.21±0.08 (6.16, 0.013)	0.16±0.06 (8.57, 0.003)
Epoch 3	-0.005±0.10 (0.002, 0.962)	0.07±0.08 (0.90, 0.344)	-6.09±4.05 (2.26, 0.133)	0.20±0.08 (6.04, 0.014)	0.09±0.08 (1.11, 0.292)

Factors influencing the relationship between Epoch 2 (Seattle-PAP Bn-CPAP) and Epochs 1 and 3 (FP Bn-CPAP). PRP, AUC and ΔP_es_ distributions were right-skewed, and the variables were analyzed on a log scale. RR had a symmetric distribution and was analyzed without transformation.

Some neonates were treated with surfactant, and some infants were administered ampicillin and gentamicin, but those treated and not treated did not differ by gestational age at birth, postnatal age at the start of study, or birth weight (S3 Table).

Adjusting for gestational age at birth, postnatal age at start of study, administration of surfactant, and administration of ampicillin and gentamicin, GEE analyses indicated that PRPs did not differ among epochs, but ΔPes were lower in Epoch 2 than during Epoch 3, and (P_es_×t) AUC per breath and per min were lower in Epoch 2 than in Epochs 1 and 3 (Table 4). The GEE analyses indicated no differences associated with gestational age at birth or time during the study periods; however, other than RR, differences were associated with postnatal age at start of study, despite the rather narrow range of times employed (44±12 h, mean±SD; range 14 to 71 h). No differences were indicated between infants treated with surfactant and those not treated.

**Table 4:** GEE model fit of PRP, ΔP_es_, RR, and AUC per breath and per min.

Response:	PRP	ΔP_es_	RR	AUC per Breath	AUC per Min
Units	cmH_2_O/min	cmH_2_O	bpm	cmH_2_O×min	cmH_2_O×min/min
Gestational Age, (wks)	0.08±0.07 (1.67, 0.196)	0.04±0.05 (0.78, 0.378)	3.06±1.75 (3.05, 0.081)	0.03±0.04 (0.32, 0.570)	0.06±0.05 (1.43, 0.232)
Postnatal age at study, (days)	0.19±0.09 (4.34, 0.037)	0.29±0.10 (7.54, 0.006)	-7.41±4.86 (2.32, 0.128)	0.35±0.15 (5.63, 0.018)	0.24±0.10 (6.29, 0.012)
Surfactant administration	-0.18±0.19 (0.85, 0.357)	-0.19±0.15 (1.62, 0.203)	0.07±6.01 (0.00, 0.991)	-0.20±0.15 (1.81, 0.179)	-0.17±0.16 (1.17, 0.280)
Ampicillin and gentamicin administration	-0.70±0.23 (9.46, 0.002)	-0.52±0.16 (10.25, 0.001)	-13.33±6.32 (4.44, 0.035)	-0.30±0.13 (5.49, 0.019)	-0.53±0.17 (9.63, 0.002)
Epoch 1	0.005±0.05 (0.01, 0.928)	0.06±0.05 (1.48, 0.224)	-4.15±3.48 (1.43, 0.232)	0.20±0.08 (5.94, 0.015)^ŧ^	0.10±0.04 (4.91, 0.027)^ŧ^
Epoch 3	0.04±0.09 (0.24, 0.621)	0.14±0.05 (6.67, 0.010)*	-6.35±4.23 (2.25, 0.133)	0.20±0.08 (6.34, 0.012)^ŧ^	0.15±0.06 (6.65, 0.010)^ŧ^

Model results are expressed as the mean difference±empirical SE of the differences. Numbers in parentheses are the Wald statistics and the associated P-values. PRP, ΔP_es_, and AUC distributions were not symmetric, and the variables were analyzed on a log scale. RR data had symmetric distributions and were analyzed without transformation. The baseline was 29.6 wks gestational age at birth, 1.84 d postnatal at start of study, no surfactant, no ampicillin or gentamicin, and Epoch 2 (Seattle-CPAP). AUC and AUC × RR were calculated as described in Table 4 and in the Methods.

PRP and RR did not differ among epochs.

*ΔPes was higher in Epoch 3 than in Epoch 2 (P<0.05).

^ŧ^AUC per breath and AUC per min were both lower (P<0.05) in Epoch 2 (Seattle-CPAP) than in Epochs 1 and 3 (FP-CPAP).

**Fig 7:**
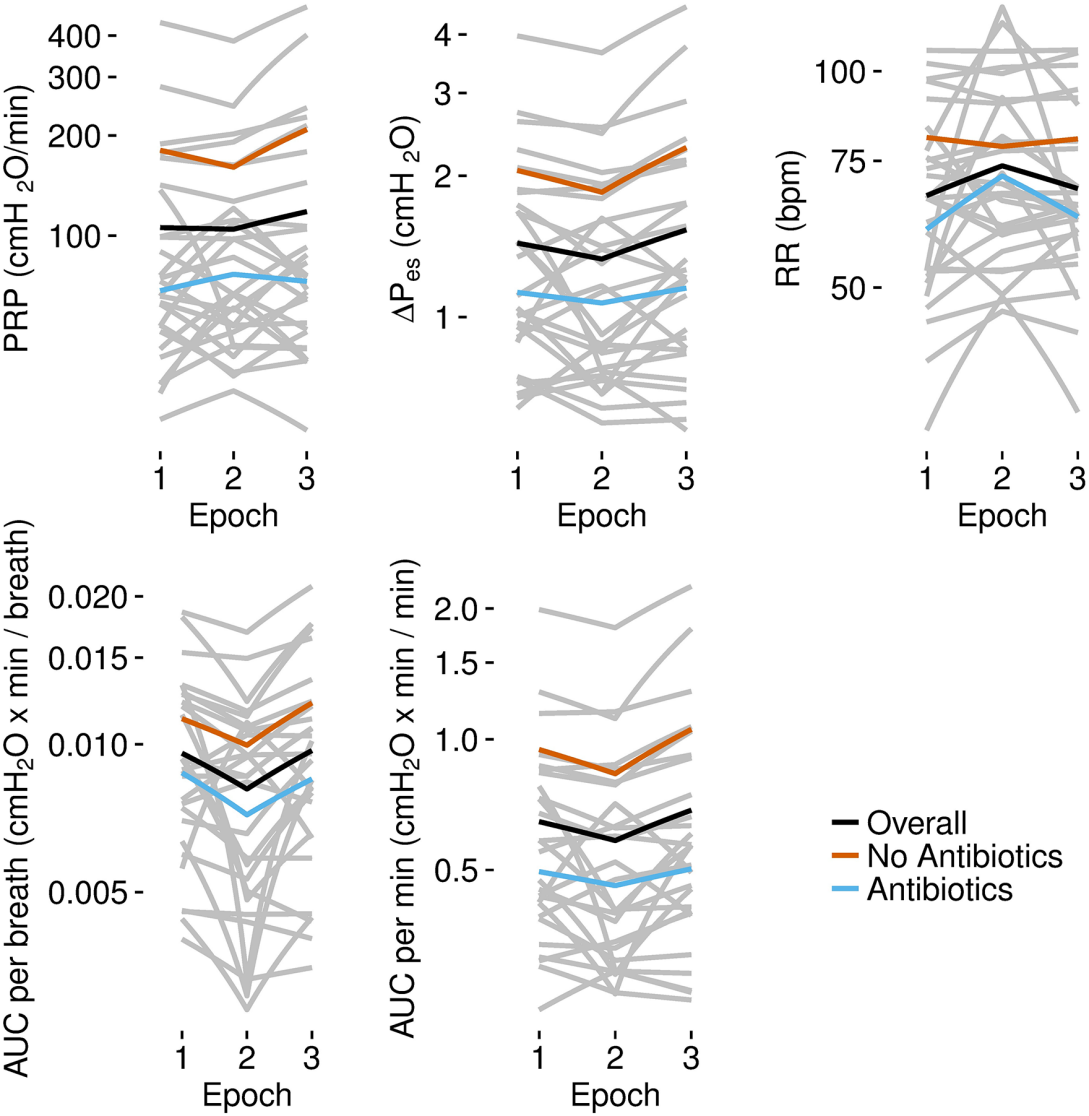
PRP, ΔP_es,_ RR, and AUCs in individual infants, aggregated by receipt of ampicillin and gentamicin. The data presented in the respective figures in gray show the median values of each infant for his or her epochs. The dark black lines are the mean values over all medians. The blue lines are the mean values of the median values for infants who were given ampicillin and gentamicin, and the red lines are the respective mean values for the infants not treated similarly.

In contrast, differences between infants treated with antibiotics and those not treated were striking. PRP, ΔP_es_, RR, and AUCs were all lower throughout all three epochs for infants who received antibiotics than in those who did not receive antibiotics (Table 5, Fig 7).

**Table 5:** Data summary by epoch and administration of antibiotics.

Variable		Epoch 1, n = 27	Epoch 2, n = 27	Epoch 3, n = 27
Numbers of data points	Overall	40 (19, 57)	22 (16, 40)	39 (24, 56)
	Ampicillin and Gentamicin			
	yes	27 (17, 40)	21 (15, 29)	33 (23, 41)
	no	59 (51, 63)	42 (16, 64)	64 (46, 65)
PRP	yes	68.3±32.9	76.5±33.9	72.9±33.3
	no	180.5±120.7	160.6±112.7	208.6±156.2
ΔP_es_	yes	1.13±0.44	1.07±0.41	1.15±0.42
	no	2.05±0.98	1.84±0.97	2.30±1.33
RR	yes	60.3±15.4	71.5±22.4	62.7±14.1
	no	80.9±21.6	78.6±21.7	80.5±22.8
AUC per breath ×10^3^	yes	8.74±3.88	7.18±3.31	8.49±3.51
	no	11.27±3.93	9.95±4.10	12.13±5.43
AUC per min	yes	0.50±0.18	0.46±0.17	0.50±0.17
	no	0.95±0.49	0.83±0.49	1.05±0.66

Numbers of data points used in the analyses expressed as medians and interquartile range (25%tile, 75%tile); summary statistics for PRP, ΔP_es_, AUC per Breath, and AUC per min are calculated as the mean±SD of patient-level medians; RR expressed as means±SD of patient-level means. Data summaries show that all response variables tend to be lower in patients who received antibiotics than in those who did not. This observation holds across all Epochs.

## Discussion

The nearly 2 to 1 female to male ratio among the patients (Table 1) may be influenced by the study design requirement that the infants be stable on FiO_2_ ≤0.30. Despite the prematurity of the neonates studied, no infants were on supplemental oxygen at 36 wks postmenstrual age or at discharge from the hospital, whichever came first. This absence of CLD in these infants is probably best attributed to the entry criteria for the studies (stable on Bn-CPAP at FiO_2_ ≤.0.30), which resulted in a patient population that was reasonably healthy, despite being born at ≤32 weeks gestation, with gestational ages at birth as low as 25 6/7ths wks and birth weights as low as 625 g (Supplemental Table 1). The implementation of noninvasive respiratory support of the infants also may have contributed to the absence of CLD in the infants studied.

The absence of striking differences in PRPs measured in neonates supported by Seattle-PAP versus conventional Bn-CPAP was at first a bit surprising, in view of the marked differences in PRPs that had been observed in preclinical studies [20]. However, analysis of the present data computed as area under the P_es_-time curve per breath and per min indicated clear differences, with neonates exhibiting lower efforts when supported by Seattle-PAP than during support by conventional Bn-CPAP. While the differences are relatively small, the infants in our study had mild lung disease, so that the effects of Seattle-CPAP on the lung would be expected to be small compared to the effects observed in studies of animals with severe respiratory distress. [12, 20].

Premature infants typically require some form of respiratory support because of hypoxemia secondary to lung disease, respiratory fatigue, or apnea. Hypoxemia in infants with severe lung disease is caused by large numbers of open, but severely under-ventilated units of the lung [30, 31]. The only way to improve oxygenation in infants with lung disease is to increase oxygen delivery to these underventilated lung units, either by administration of supplemental oxygen or by improving ventilation. Improvements in ventilation can be attained by application of CPAP, administration of exogenous surfactant, or by mechanical ventilation with positive end expiratory pressure (PEEP)

CPAP recruits open but poorly ventilated lung units, increases oxygenation, increases lung compliance (C_L_), and increases lung volume [31, 32], all of which would allow supported infants to expend less effort to maintain oxygenation. Studies on lambs with respiratory distress found that Bn-CPAP resulted in better lung recruitment, with attendant larger increases in lung volume and oxygenation than did Vn-CPAP [12], while data from rabbits with RDS from saline lavage showed better oxygenation and markedly lower PRP with Seattle-PAP than with standard Bn-CPAP [20]. Additionally, data from studies in human infants showed that infants treated with Bn-CPAP were better oxygenated than were those treated with Vn-CPAP [13].

The poorly ventilated parts of the lung in preterm infants and animals with RDS do not comprise a uniform compartment, with all lung units having roughly the same airway resistance (R_AW_), compliance (C_L_), or time constant (TC), but are a very heterogeneous group with a wide variety of TCs. Consequently, methods of respiratory support that offer ventilation at a variety of frequencies might be better at recruiting severely under-ventilated lung units. If so, the varying amplitudes and frequencies of pressures generated by Seattle-PAP might be better at lung recruitment, resulting in higher lung volumes, better oxygenation, and lower efforts of breathing [20]. Interestingly, we found that RRs were slightly higher in Epoch 2 than in 1 and 3. The reasons for the slightly higher RRs are not obvious from the evidence presently available, but might be attributed to modest levels of over-inflation. Since the infants were breathing spontaneously, and thus setting their own RRs, reductions in C_L_ and possibly R_AW_ as well, resulting from increased lung volumes, might have led to their breathing at higher RRs [33].

In the case of severe lung disease, Bn-CPAP improves ventilation to poorly ventilated lung units, allowing supported infants to expend less effort to maintain oxygenation. In the case of apnea, Bn-CPAP helps prevent obstructive apnea by supporting extra-pulmonary airways and for central apnea aids in the generation or transmission of neural drive to compensate for defects in ribcage support for generation of pleural pressure swings, and/or by fatigue of the respiratory muscles, principally the diaphragm [34]. Respiratory failure is defined biochemically and clinically as either hypoxemia and/or hypercarbia, but fundamentally, and functionally, respiratory muscle fatigue plays a major role in failure of CPAP support. Respiratory muscle fatigue has been attributed to interactions of a number of factors [35, 36], with a detailed investigation of these factors supporting the interpretation that the key determinant of fatigue is whether consumption of energy or oxygen by a muscle exceeds the existing stores plus the ongoing resupply by blood flow.

Bellemare and Grassino related respiratory fatigue of the diaphragm to a parameter they termed the tension time index of the diaphragm (TT_di_). The TT_di_ is determined by the product of the ratio of transdiaphragmatic pressure per breath to the maximal pressure that can be attained by the individual (P_di_/Pdi_max_) times the duty cycle of the diaphragm (T_i_/T_tot_). Although other factors can contribute to muscle fatigue, the T_i_/T_tot_ reflects the fraction of the breathing cycle that is spent in relaxation (T_tot_-T_i_), the time period in which blood flow to support resupply to the muscle of oxygen and metabolic substrates occurs [24]. In their studies, diaphragmatic fatigue was observed when TT_di_ exceeded an apparently critical value of 0.15. Such a value would arise from, for example, half maximal pressure generation during inspiration and 30% of breath cycle time spent in inspiration, or vice versa, with 30% of maximum force generation with half of the cycle time in inspiration.

Measurements of Pdi_max_ in preterm neonates would seem to be impractical, if not unattainable, at the present time, but the respective Pdi_max_ for each individual neonate is likely to remain constant for that neonate during the six hour course of our study. The composite estimates calculated in the present studies as AUC per breath, and especially AUC per min, thus are reasonable candidates as predictors of inspiratory muscle fatigue. The neonates studied in the present efforts were not sedated and at times became rather active. These activities, while representative of realistic conditions encountered clinically, obviously complicated efforts to collect ‘clean’ data on respiratory efforts. In more fully resourced NICUs, the more proximate question is whether Bn-CPAP with Seattle-PAP would provide greater respiratory support than is provided by conventional Bn-CPAP, thereby decreasing failure rates and CLD. These data will have important longer term applicability in LMICs, because the results define the effects to be expected, as adoption of these and other therapies that improve survival of prematurely born infants are implemented. In LMICs in which constraints on health care budgets can be quite severe, improvements in survival of infants born prematurely will be received much more favorably if improvement in survival is not accompanied by increases in chronic conditions, such as BPD, that are costly to manage. Implementation of Bn-CPAP in LMIC settings should improve long term outcomes as well as survival [37]. Resolving the extent to which Seattle-PAP would improve outcomes in LMICs even further will require direct testing in actual care settings.

Possibly the most surprising findings in the results of the present study are the uniformly lower PRP, ΔPes, RR, AUC per breath, and AUC per min of neonates receiving gentamicin and ampicillin than in infants not receiving these antibiotics (Tables 4 and 5 and Fig 7). The present studies do not address possible effects of the drugs themselves. In general, ampicillin and gentamicin were administered to neonates born to mothers with suspected maternal infections, and maternal inflammatory responses can accelerate maturation of fetal lung structure and surfactant metabolism [38]. Reports of primary cardiovascular effects of gentamicin [39, 40] suggest that comparable effects on effort of breathing should be considered, but at present, the weight of evidence suggests stronger correlations with the effects of maternal inflammatory responses on maturation of lung function.

## Conclusions

The efficacy, safety, and practical advantages of Bn-CPAP in LMICs have been demonstrated [5, 14, 16, 41]. The results of the present studies show that prematurely born neonates with minimal parenchymal lung disease supported by Bn-CPAP exhibit lower effort of spontaneous breathing with Seattle-PAP than with conventional Bn-CPAP. The major limitations of the present studies include the lack of blinding and the lack of randomization, restriction to studies of a relatively healthy subgroup of newborns requiring respiratory support, and the technical issues, primarily inflation of the esophageal catheter and infant body motions, that resulted in our inability to include data from a larger fraction of the infants studied than one would prefer. Whether the differences between Seattle-PAP and conventional Bn-CPAP, such as observed in the present studies, prove to be associated with lower morbidity, mortality, and cost of care for neonates born prematurely in LMIC as well as in economically developed countries will require additional investigation.

## Supporting information

S1 Table Detailed patient demographics. Detailed demographic data are presented for each patient in the study.
(DOCX)

S2 Table Device-specific data. Data are presented for each study patient for tub depth, gas flow rates, and FiO2 for each epoch.
(DOCX)

S3 Table Demographics of study infants summarized by surfactant and antibiotics administration. Data on patient demographics compared between patients treated with ampicillin and gentamicin and those not treated similarly.
(DOCX)

S1 Fig Trend statement trend checklist. (DOCX)

S1 Text H-29620 IRB approved BRAIN summary. This is the protocol approved by the Baylor/TCM IRB.
(PDF)

S2 Text H-29620 IRB approval letter. This is a copy of the letter of approval issued by the Baylor/TCM IRB.
(PDF)

S3 Text H-29620 IRB approved ICF. This is a copy of the consent form for the study, as approved by the Baylor/TCH IRB.
(PDF)
