# Supplementary material for: Short term evaluation of respiratory effort by premature infants supported with bubble nasal continuous airway pressure using Seattle-PAP and a standard bubble device
Source: PLoS One. 2018 Mar 28;13(3):e0193807. doi: 10.1371/journal.pone.0193807 (PMC5874011; doi:10.1371/journal.pone.0193807)
Supplement: S1 Table — Detailed demographic data are presented for each patient in the study. (DOCX) [file pone.0193807.s001.docx]

S1 Table. Detailed Patient Demographics

| Pt  # | Gender | Ethnicity | Race | Birth weight (g) | Catheter placement (h) | G.A.at birth (wks) | ANCS | Amp  Gent | Caffeine | Surf. | Off study reason |
| --- | --- | --- | --- | --- | --- | --- | --- | --- | --- | --- | --- |
| 001 | M | 0 | White | 1050 | 37 | 27.1 | y | y | y | y | 36 |
| 002 | M | 0 | White | 1265 | 66 | 32.3 | y | n | y | y | dc |
| 003 | M | 0 | B/AA | 1005 | 25 | 29.1 | y | n | y | y | 36 |
| 004 | F | 0 | White | 625 | 41 | 28.0 | y | n | y | n | 36 |
| 005 | F | 0 | B/AA | 1410 | 43 | 31.6 | y | n | y | n | dc |
| 006 | F | 0 | B/AA | 1151 | 45 | 28.4 | y | y | y | y | dc |
| 007 | M | 0 | White | 1445 | 52 | 28.7 | y | n | y | y | dc |
| 008 | F | 0 | B/AA | 1290 | 41 | 31.3 | y | n | y | n | dc |
| 009 | M | 0 | White | 1875 | 42 | 30.9 | n | y | y | y | dc |
| 010 | F | 0 | B/AA | 1364 | 34 | 31.0 | y | y | y | n | 36 |
| 011 | M | 0 | White | 1160 | 60 | 28.4 | y | y | y | y | dc |
| 012 | F | 0 | B/AA | 1610 | 24 | 31.4 | y | y | y | y | dc |
| 013 | M | 0 | White | 975 | 43 | 26.0 | y | y | y | y | 36 |
| 014 | F | 0 | White | 930 | 60 | 25.6 | y | y | y | y | 36 |
| 015 | M | H | White | 1361 | 41 | 27.6 | y | y | y | y | 36 |
| 016 | F | 0 | White | 1446 | 71 | 29.7 | y | y | y | y | dc |
| 017 | F | 0 | White | 1345 | 64 | 29.3 | n | y | y | n | 36 |
| 018 | F | H | White | 970 | 53 | 27.1 | y | n | y | y | 36 |
| 019 | F | 0 | White | 1248 | 14 | 29.1 | y | y | y | y | 36 |
| 020 | F | 0 | White | 1673 | 57 | 31.3 | y | n | y | y | 36 |
| 021 | M | 0 | B/AA | 1500 | 49 | 31.0 | y | y | y | n | dc |
| 022 | F | 0 | B/AA | 1304 | 34 | 30.7 | y | y | y | n | dc |
| 023 | M | 0 | B/AA | 1155 | 49 | 27.6 | y | y | y | n | dc |
| 024 | M | H | White | 1009 | 47 | 27.6 | y | n | y | y | dc |
| 025 | F | H | White | 1250 | 37 | 31.4 | y | n | y | y | 36 |
| 026 | M | H | White | 1155 | 36 | 28.7 | y | y | y | n | 36 |
| 027 | F | H | White | 1110 | 60 | 28.7 | y | y | y | n | 36 |
| 028 | F | H | White | 1420 | 50 | 29.1 | y | y | n | n | dc |
| 029 | F | 0 | White | 904 | 40 | 27.0 | y | y | y | n | 36 |
| 030 | F | H | White | 1440 | 35 | 30.6 | y | y | y | n | dc |
| 031 | F | H | White | 1605 | 45 | 31.1 | y | n | n | n | 36 |
| 032 | F | 0 | Asian | 1641 | 47 | 30.9 | y | y | y | n | 36 |
| 033 | F | 0 | White | 1471 | 47 | 30.7 | y | n | y | y | 36 |
| 034 | F | H | White | 1744 | 23 | 30.7 | y | y | y | y | dc |
| 035 | M | H | White | 1610 | 48 | 31.7 | y | y | y | n | dc |
| 036 | F | 0 | White | 1219 | 61 | 28.1 | y | n | y | y | 36 |
| 037 | F | 0 | B/AA | 1450 | 37 | 31.3 | y | n | n | n | dc |
| 038 | F | 0 | White | 961 | 42 | 29.4 | y | y | y | y | 36 |
| 039 | F | 0 | B/AA | 1179 | 51 | 27.3 | y | y | y | y | 36 |
| 040 | M | 0 | White | 1599 | 59 | 29.9 | y | y | n | y | dc |

Ethnicity Hispanic (H), non-Hispanic (0); Race B/AA - Black African American; GA - Gestational age; ANCS, antenatal corticosteroid administration to mother; Amp Gent - antibiotics ampicillin and gentamicin administered to infant; yes (y), no (n).
