# Supplementary material for: Short term evaluation of respiratory effort by premature infants supported with bubble nasal continuous airway pressure using Seattle-PAP and a standard bubble device
Source: PLoS One. 2018 Mar 28;13(3):e0193807. doi: 10.1371/journal.pone.0193807 (PMC5874011; doi:10.1371/journal.pone.0193807)
Supplement: S2 Table — Data are presented for each study patient for tub depth, gas flow rates, and FiO2 for each epoch. (DOCX) [file pone.0193807.s002.docx]

S2 Table. Device-Specific Data

|  | Epoch 1 | | | Epoch 2 | | | Epoch 3 | | |
| --- | --- | --- | --- | --- | --- | --- | --- | --- | --- |
| Study ID | *Tube depth  (cmH_2_O) | Flow rate  (l/min) | FiO_2_ | Tube depth  (cmH2O) | Flow rate  (l/min) | FiO_2_ | Tube depth  (cmH_2_O) | Flow rate  (l/min) | FiO_2_ |
| 001 | 8 | 6 | 0.29-0.27 | 8 | 6 | 0.27-0.25 | 8 | 6 | 0.25-0.26 |
| 002 | 6 | 6 | 0.26-0.25 | 6 | 6 | 0.25-0.26 | 6 | 6 | 0.25 |
| 003 | 8 | 8 | 0.21 | 8 | 8 | 0.21 | 8 | 8 | 0.21 |
| 004 | 6 | 6 | 0.21 | 6 | 6-7 | 0.21 | 6 | 7 | 0.21 |
| 005 | 8 | 8 | 0.21 | 8 | 8 | 0.21 | 8 | 8 | 0.21 |
| 006 | 8 | 8 | 0.21 | 8 | 8 | 0.21 | 8 | 8 | 0.21 |
| 007 | 6 | 8 | 0.21 | 6 | 8 | 0.21 | 6 | 8 | 0.21 |
| 008 | 6 | 8 | 0.33-0.30 | 6 | 8 | 0.30-0.28 | 6 | 8 | 0.28 |
| 009 | 6-7 | 8 | 0.24-0.21 | 6 | 8 | 0.24-0.21 | 6 | 8 | 0.21 |
| 010 | 8 | 8 | 0.21 | 8 | 8 | 0.21 | 8 | 8 | 0.21 |
| 011 | 8 | 8 | 0.21 | 8 | 8 | 0.21 | 8 | 8 | 0.21 |
| 012 | 8 | 8 | 0.21 | 8 | 8 | 0.21 | 8 | 8 | 0.21 |
| 013 | 8 | 8 | 0.21 | 8 | 8 | 0.21 | 8 | 8 | 0.21 |
| 014 | 8 | 8 | 0.21 | 8 | 8 | 0.21 | 8 | 8 | 0.21 |
| 015 | 6 | 7 | 0.21 | 6 | 7 | 0.21 | 6 | 7 | 0.21 |
| 016 | 8 | 8 | 0.21 | 8 | 8 | 0.21 | 8 | 8 | 0.21 |
| 017 | 6 | 8 | 0.21 | 6 | 8 | 0.21 | 6 | 8 | 0.21 |
| 018 | 6 | 8 | 0.21 | 6 | 8 | 0.21 | 6 | 8 | 0.21 |
| 019 | 8 | 8 | 0.21 | 8 | 8 | 0.21 | 8 | 8 | 0.21 |
| 020 | 6-5 | 8 | 0.21 | 5 | 8 | 0.21 | 5 | 8 | 0.21 |
| 021 | 8 | 8 | 0.21 | 8 | 8 | 0.21 | 8 | 8 | 0.21 |
| 022 | 6 | 7 | 0.21 | 6 | 7 | 0.21 | 6 | 8 | 0.21 |
| 023 | 8 | 8 | 0.21 | 6 | 8 | 0.21 | 8 | 8 | 0.21 |
| 024 | 6 | 7 | 0.21 | 6 | 7 | 0.21 | 6 | 7 | 0.21 |
| 025 | 6 | 7 | 0.21 | 6 | 7 | 0.21 | 6 | 7 | 0.21 |
| 026 | 6 | 8 | 0.21 | 6 | 8 | 0.21-0.96 | 6 | 8 | 0.21 |
| 027 | 6 | 7 | 0.21 | 6 | 7 | 0.21 | 6 | 7 | 0.21 |
| 028 | 6 | 8 | 0.21 | 6 | 8 | 0.21 | 6 | 8 | 0.21 |
| 029 | 6 | 7 | 0.24 | 6 | 7 | 0.24 | 6 | 7 | 0.24 |
| 030 | 6 | 8 | 0.22-0.21 | 6 | 8 | 0.21 | 6 | 8 | 0.21 |
| 031 | 8 | 8 | 0.21 | 8 | 8 | 0.21 | 8 | 8 | 0.21 |
| 032 | 8 | 7 | 0.21 | 8 | 7 | 0.21 | 8 | 7 | 0.21 |
| 033 | 6 | 7 | 0.21 | 6 | 7 | 0.21 | 6 | 7 | 0.21 |
| 034 | 6 | 8 | 0.21 | 6 | 8 | 0.21 | 6 | 8 | 0.21 |
| 035 | 6 | 7 | 0.21 | 6 | 7 | 0.21 | 6 | 7 | 0.21 |
| 036 | 6 | 8 | 0.21 | 6 | 8 | 0.21 | 6 | 8 | 0.21 |
| 037 | 6 | 7 | 0.29 | 8 | 7 | 0.29-0.27 | 8 | 7 | 0.27 |
| 038 | 6 | 7 | 0.21 | 6 | 7 | 0.21 | 6 | 7 | 0.21 |
| 039 | 6 | 8 | 0.26 | 6 | 8 | 0.26 | 6 | 8 | 0.26 |
| 040 | 5 | 8 | 0.21 | 5 | 8 | 0.21 | 5 | 8 | 0.21 |

*Minimum and maximum values in a range of values are separated by a hyphen.
