# Supplementary material for: Short term evaluation of respiratory effort by premature infants supported with bubble nasal continuous airway pressure using Seattle-PAP and a standard bubble device
Source: PLoS One. 2018 Mar 28;13(3):e0193807. doi: 10.1371/journal.pone.0193807 (PMC5874011; doi:10.1371/journal.pone.0193807)
Supplement: S3 Table — Data on patient demographics compared between patients treated with ampicillin and gentamicin and those not treated similarly. (DOCX) [file pone.0193807.s003.docx]

S3 Table. Demographics of Study Infants Summarized by Surfactant and Antibiotics Administration

|  | Surfactant | | | Ampicillin and Gentamicin | | |
| --- | --- | --- | --- | --- | --- | --- |
|  | Yes, n=18 | No, n=9 | *t*, P | Yes, n=18 | No, n=9 | *t*, P |
| Gestational age at birth, (wks) | 29.2±1.7 | 30.5±1.4 | -1.94, 0.06 | 29.3±1.8 | 30.2±1.5 | -1.32, 0.20 |
| Age at start of study, (h) | 45.5±15.7 | 41.6±6.3 | 0.93, 0.36 | 44.2±14.7 | 44.1±10.9 | -0.02, 0.98 |
| Birth weight, (g) | 1304±273 | 1397±171 | -1.07, 0.29 | 1337±254 | 1330±239 | 0.07, 0.94 |

Data are mean+SD for infants treated or not with surfactant or ampicillin and gentamicin and were compared statistically by unpaired t-tests.
