# Supplementary material for: Short term evaluation of respiratory effort by premature infants supported with bubble nasal continuous airway pressure using Seattle-PAP and a standard bubble device
Source: PLoS One. 2018 Mar 28;13(3):e0193807. doi: 10.1371/journal.pone.0193807 (PMC5874011; doi:10.1371/journal.pone.0193807)
Supplement: S1 Text — This is the protocol approved by the Baylor/TCM IRB. (PDF) [file pone.0193807.s005.PDF]

✓Compare

Institutional Review Board for Baylor College of Medicine and Affiliated Hospitals

Protocol Number: H-29620
Status: Approved
Initial Submit Date: 3/14/2013
Approval Period: 2/6/2014 - 2/5/2015

Section Aa: Title & PI

A1. Protocol Title

A STUDY TO EVALUATE THE EFFICACY OF SEATTLE-PAP FOR THE RESPIRATORY SUPPORT OF PREMATURE INFANTS

A2. Principal Investigator

Name:	STEPHEN WELTY	Phone:	832-826-1380
Id:	017690	Fax:	832-825-2799
Department:	PEDIATRICS: NEWBORN	Email:	welty@bcm.tmc.edu
Center:		Mail Stn:	BCM320

A3. Administrative Contact

Name:	SUPRIYA PARIKH	Phone:	832-824-3379
Id:	178600	Fax:	
		Email:	supriyap@bcm.tmc.edu
		Mail Stn:	BCM320

A3a. Financial Conflict of Interest

Does the investigator have a financial interest in any non-Baylor sponsor or funding source for this research?
No

Section Ab: General Information

A4. Co-Investigators

Name:	ALFRED L GEST	Phone:	832-826-1380
Id:	016072	Fax:	
Department:	PEDIATRICS: NEWBORN	Email:	gest@bcm.edu
Center:		Mail Stn:	BCM320

Name:	GEORGE T MANDY	Phone:	832-826-1380
Id:	035912	Fax:	
Department:	PEDIATRICS: NEWBORN	Email:	gmandy@bcm.edu
Center:		Mail Stn:	BCM320

Name:	UMA RAMAMURTHY	Phone:	713-798-8920
Id:	173559	Fax:	713-798-2816
Department:	PEDIATRICS: RESEARCH RESOURCE OFFICE	Email:	uramamur@bcm.tmc.edu
Center:		Mail Stn:	BCM122

Name: CHARLES G. MINARD
Id: 174095
Department: PEDIATRICS: RESEARCH RESOURCE OFFICE
Center:
Phone: 713-798-2353
Fax:
Email: minard@bcm.tmc.edu
Mail Stn: BCM122

Name: CRAIG RUSIN
Id: 175172
Department: PEDIATRICS: CARDIOLOGY
Center:
Phone: 832-826-6230
Fax:
Email: crusin@bcm.tmc.edu
Mail Stn: BCM320

Name: CHRISTOPHER HOWARD
Id: Non-Baylor
Institution: Seattle Children's Research Institute
Address: 1900 Ninth Avenue, Seattle WA 98101
Phone: 206 884-1271
Fax:
Email: christopher.howard@seattlechildrens.org

Name: CHARLES SMITH
Id: Non-Baylor
Institution: Seattle Children's Research Institute
Address: 1900 Ninth Avenue, C9S-9, Seattle WA, 98101
Phone: 206 884-1271
Fax:
Email: charles.smith@seattlechildrens.org

Name: PETER RICHARDSON
Id: Non-Baylor
Institution: Seattle Children's Research Institute
Address: 1900 Ninth Avenue, Seattle WA 98101
Phone: 206 884-7302
Fax:
Email: peter.richardson@seattlechildrens.org

Name: THOMAS HANSEN
Id: Non-Baylor
Institution: Seattle Children's Hospital
Address: 4800 Sand Point Way NE, Seattle, WA 98105
Phone: 206 987-2001
Fax:
Email: hansen@seattlechildrens.org

Name: JONATHAN POLI
Id: Non-Baylor
Institution: Seattle Children's Research Institute
Address: 1900 Ninth Avenue, Seattle WA 98101
Phone: 206 884-7646
Fax:
Email: jonathan.poli@seattlechildrens.org

A5. Funding Source:

Organization: SEATTLE CHILDREN'S HOSPITAL RESEARCH FOUNDATION

A6a. Institutions where work will be performed:

Seattle Children's Hospital - Washington
TCH: Texas Children's Hospital

A6b. Research will be conducted outside of the United States:

Country:
Facility/Institution:
Contact/Investigator:
Phone Number:

If documentation of assurances has not been sent to the Office of Research, please explain:

A7. Research Category:

Section B: Exempt Request

B. Exempt From IRB Review

Not Applicable

Section C: Background

Babies born prematurely the infant is at high risk for mortality and morbidity. Advances in acute supportive care have improved survival in infants less than 1500 grams, but these improvement in survival rates have plateaued over the last decade. Survival to discharge in this patient population is no longer the dominant goal in research efforts, which are now directed more toward decreasing the rates of complications in this vulnerable patient group. One of the most common complications of prematurity is the development of chronic lung disease, termed bronchopulmonary dysplasia (BPD). The exact pathophysiology of BPD has not been elucidated entirely, but the dominant pathophysiology is probably related to supportive care employed for infants with underdeveloped lungs, which can create lung injury and arrest alveolar development. Respiratory support for very low birth weight (VLBW) (< 1500 grams) infants has historically included mechanical ventilation, continuous positive airway pressure (CPAP) or no positive pressure, +/- supplemental oxygen. Mechanical ventilation damages the lung as it is non-physiologic, and no positive pressure is untenable because the respiratory system is immature to the extent that lung collapse ensues even when the lung mechanics are normal. CPAP assists spontaneously breathing infants by preventing lung collapse, and nasal CPAP, provided through nasal prongs, affords the additional benefit of avoiding the damaging effects of endotracheal intubation. Establishing the system pressure by having the system gas exit through a tube submerged in water, in addition to being much cheaper and easier to regulate than with a mechanical ventilator, provides additional benefits through the small pressure oscillations caused by the effects of the bubbling of the gas leaving the tube immersed in the water bath. Thus, providing respiratory support by bubble nasal CPAP (Bn-CPAP) in infants born at less than 30-32 weeks gestational age is being used by a growing number of NICUs in the US and around the world. Unfortunately, there is a significant failure rate for nasal CPAP, failure being the need for intubation for respiratory failure or significant apnea. Thus, there is a need for innovative new approaches for non-invasive respiratory support in premature infants to reduce complications of prematurity such as BPD. In addition, the great majority of the almost one million neonates who die each year due to respiratory insufficiency arising from their premature birth occur in low and middle-income countries (LMIC) and other resource-limited health care settings, in which intubation and mechanical ventilation are not available.

Colleagues from Seattle Children's Hospital have designed and developed a simple and inexpensive respiratory support device (Seattle-PAP) that is safe and effective in preclinical trials. The design is such that instead of the expiratory limb of the Bn-CPAP going straight into a column of water (0 degree angle), the expiratory limb is at the same depth so that the airway pressure is identical (to that set with Bn-CPAP) but gas flow exits through a tube bent to 135 degrees, up toward the surface of the water. In a model in vitro, this modification increases airway pressures oscillations in a model lung relative to standard Bn-NCPAP. Seattle-PAP has been studied in experimental animals and found to provide superior support for the respiratory system. The endpoints assessed physiologically include:

1. Partial pressure of O2 in arterial blood (PaO2) with Seattle-PAP being higher than standard Bn-CPAP.

2. No differences in PaCO2 between groups.

3. Markedly lower work of breathing as assessed by a Pressure Rate Product.

Based on this study we propose to test the hypothesis that respiratory support with Seattle-PAP exhibits lower estimates of work of breathing in VLBW infants than standard Bn-CPAP.

No long term beneficial effect is expected to be observed in the infants to be studied in the trials proposed. Seattle-PAP may provide greater support than Bn-CPAP, and the physiological data assessed are designed to test the hypothesis of enhanced respiratory support, but we have made no provisions to continue the experimental device in the present trial, as Bn-CPAP is presently the Texas Children's Hospital (TCH) nursery standard for clinical care.

The data to be obtained in the studies proposed are essential to demonstrating device performance in humans, and may impact the following efficacy trial. The longer term goals of the studies planned are to improve non-invasive respiratory support, limit acute lung injury, and diminish chronic respiratory morbidity in a population highly susceptible to these complications of prematurity. The studies proposed are logical in sequence, and the results to be obtained are essential to determine whether Seattle-PAP has the potential to reduce the incidence of BPD and/or chronic respiratory morbidity in highly resourced NICUs and to prevent perhaps hundreds of thousands of neonatal deaths due to respiratory distress each year in resource-limited settings.

Section D: Purpose and Objectives

We propose to test the hypothesis that Seattle bubble nasal continuous positive airway pressure (Seattle-PAP) supports respiratory physiology in very low birth weight (VLBW) infants more effectively than standard bubble nasal continuous positive airway pressure

(Bn-CPAP).

The primary outcome variable is work of breathing (WOB) over a two hour period, estimated from pressure-rate products, which are assessed with 6 Fr (2 mm) catheters placed in the distal esophagus for monitoring esophageal pressures (Pes), thereby estimating changes in pleural pressures during breath cycles. The following endpoints would also be assessed: Oxygen saturations and Fraction of Inspired Oxygen (FiO2) needed to keep saturations in acceptable ranges, Heart Rates (HR), transcutaneous carbon dioxide (TcPCO2), and respiratory rates throughout the 6 hour study period. The primary outcome measure will be evaluated during the last 15 minutes of each 2 hour study period. If the subject needs clinical interventions during this period, the closest preceding 15 minutes will be used for analysis.

Objective determination of when an infant requires more or less respiratory support is difficult, but measurements of pressure-rate products as estimates of work of breathing, using esophageal catheters, can estimate an infant's respiratory effort. However, objective, simple-to-use, low cost, and non-invasive methods and tools to determine an infant's respiratory effort do not exist currently.

This study also is designed to test the hypothesis that infants' chest and abdominal movements can be assessed quantitatively from video images in ways that can be correlated with intrathoracic pressures, as measured with esophageal catheters. The video procedures will be implemented when and if the process can be set up before the enrollment is completed.

Section E: Protocol Risks/Subjects

E1. Risk Category

Category 2: Research involving greater than minimal risk, but presenting the prospect of direct benefit to the individual subjects.

E2. Subjects

Gender:
Both

Age:
Premature Infant (<37 weeks gestational age)

Ethnicity:
All Ethnicities

Primary Language:
English, Spanish

Groups to be recruited will include:
Patients

Vulnerable populations to be recruited as subjects:
Children

Vulnerable populations require special protections. How will you obtain informed consent, protect subject confidentiality, and prevent undue coercion?
Informed consent will be obtained from one or both parents of an eligible infant. Data will be entered prospectively during the study period, while demographics will be abstracted from the chart. All data will be recorded and coded. This will be explained to parents and they will have the right to withdraw from the study at any time.

E3. Pregnant woman/fetus

Will pregnant women be enrolled in the research?
No

E4. Neonates

Will neonates be enrolled in the research?
No

E5. Children

Will children be enrolled in the research?

Yes

Section F: Design/Procedure

F1. Design

Select one category that most adequately describes your research:

i) Device, Phase I, Single Center

Discuss the research design including but not limited to such issues as: probability of group assignment, potential for subject to be randomized to placebo group, use of control subjects, etc.

This is a single site enrollment study with an enrollment target of 40 subjects. Texas Children's Hospital/Baylor College of Medicine will serve as the primary enrollment site.

Seattle Children’s Hospital will provide Seattle-PAP and the comparator F&P BC 153-10 Bn-CPAP System (60 of each device). Seattle-PAP is provided under a 510(k) exemption; however, data may need to be submitted to US or international regulatory bodies in the future.

This is a pilot study in which each infant will serve as their own control. Study procedures will commence after informed consent is obtained and eligibility is confirmed. The study consists of 3 periods: (1) Bn-CPAP (~120 minutes) (2) Seattle-CPAP (~120 minutes) (3) Bn-CPAP (~ 120 minutes).

The subject’s participation ends at the end of the 6 hour period. However, the study team will follow the subjects for targeted adverse events for 28 days from end of study AND 36 weeks gestational age OR discharge (whichever occurs first).

Inclusion Criteria:

1. Admitted to a bed at Pavilion for Women (or beds monitored by SickBay)

2. Less than or equal to 32 weeks gestational age

3. 6 to 72 hours of life (this parameter is chosen because this will be the time frame of subsequent non-inferiority trial).

4. Stable on standard bubble nasal CPAP (Bn-CPAP) defined as having less that 6 apneic spells per shift and requiring only tactile stimulation for treatment

5. On less that equal to 8 cm H20 on Bn-CPAP

6. Less than or equal to 30% oxygen requirement (this is chosen because if it is greater than 30% the patient is likely to have evolving hyaline membrane disease which is likely to dominate the serial measurements made and the effects of the device will be difficult to detect).

7. Informed Consent

Exclusion Criteria:

1. Congenital anomalies or suspected chromosomal abnormalities

F2. Procedure

The study measurements will be recorded continuously over each of the three periods (outlined in section F1) of 2 hours for a total of 6 hours. If reasonable clinically, routine nursing care should be conducted in the first 15 min of each period of 2 h; however, patient care as per normal is the prime directive.

Study-Specific Monitoring Equipment:

Upon verification of subject eligibility the DAta ReCording Instrument (DARCI 1, DARCI 2), the Video Recording System, and TcPCo2 monitor will be set up.

To address the growing demand for continuous data recording during clinical trials, SCRI and Intellectual Ventures Labs (IV Lab) have created a rugged data recording instrument (DARCI) that will be used to monitor and record, and, with further development, transmit airway pressure (PAW) data continuously. In order to complete this data collection, DARCI will record the pressure present

at the junction between the breathing circuit and the nasal prongs. DARCI is designed to be easy to use by a trained study coordinator and operates in three different modes – Enrollment (the act of setting up the device for a new test), Recording (the act of collecting data from the Device Under Test [DUT]), and Download (the act of transmitting and storing the data collected onto a PC). The esophageal catheters are commercially available and will be supplied by the Sponsor. The catheter will transmit pressures to DARCI which will transmit the transpulmonary pressure data to Sickbay for the study duration.

The Video Recording System will be installed and activated when available. When the video recording has occurred, the DVR data will be downloaded to a hard disk and delivered to Dr. Rusin for storage. Videos of the infants will be de-identified through the use of a standard phototherapy mask.

The additional monitoring equipment, a TcPCO2 monitor, will be set up and connected to the infant. The management of the oxygen administration and monitoring is per subjects' standard of care.

The physiological data described below will be captured in Dr. Rusin's database (SickBay) automatically as per H-28829 for the 6 hour study period.

• Heart rate • Respiratory rate • Blood pressure • SPO2 via Pulse oximetry. TCH patients are managed to keep the pulse oximetry between 90 and 95%. This range is evidence-based and is usually attained by respiratory support consisting of a combination of nasal CPAP (range 5-8 cm H2O) and FiO2. If the infant is on Bn-CPAP, then we would not expect an improvement, so the device should have no effect on percent saturation. The infant will still qualify for enrollment, as the other endpoints (TcPCO2 and pressure rate products) may indicate a difference of the new device, and the data will address whether Seattle-PAP leads to a deterioration. • TcPCO2, via transcutaneous PCO2 monitors. • ECG • Chest impedance pneumography • Airway and esophageal pressures via DARCI 1 and 2, respectively

The data points described below will also be captured in for the 6 hour study period:

• Demographics • Concomitant medication • Serious and non-serious adverse events • Start and Stop times of Bn-CPAP and Seattle PAP • FiO2, tube depth, gas flow rate

Study Start Time (T=0):

Once SickBay is capturing data from all the monitoring equipment, the PI/Co-I/respiratory therapist will place the esophageal catheter. Once the esophageal catheter is placed the CRA will verify that SickBay is capturing data transmitted by DARCI 2. Data collection for this study starts (T=0) once all the systems used to monitor the patient and airway and esophageal pressures are verified and working as required.

STUDY TIMEPOINTS:

See schedule of events attached in Section S.

I. Fisher-Paykel Bn-CPAP (0-2 Hours):

At T=0 (+ 5 minutes) the Respiratory Therapist will switch the patient from TCH's standard form of Bn-CPAP to the commercial Bn-CPAP Fisher-Paykel device (F&P Bn-CPAP).

II. Seattle-PAP (2-4 Hours):

At 120 minutes (+ 5 minutes), the Respiratory Therapist will switch the patient from F&P Bn-CPAP to Seattle-PAP for the following 2 hours (study period hours 2 to 4).

III. F&P Bn-CPAP (4-6 Hours):

At 240 minutes (+ 5 minutes) the Respiratory Therapist will switch the patient back to F&P Bn-CPAP.

IV. End of Study:

At 360 minutes (+ 5 minutes) the Respiratory Therapist will switch the patient back to TCH Bn-CPAP from F&P Bn-CPAP

V. Follow Up:

Follow up AE/SAE (Adverse Event/Serious Adverse Event) data collection will be performed at 28 days after enrollment, and at 36 weeks gestational age or discharge whichever is earliest.

Rationale for the study time points is as follows:

In this study, the infants will be observed continuously and closely for the entire time the infants are being assessed for the work of breathing. Unexpected observations will be reported and evaluated by the DSMB, sponsor, and IRB, as applicable. This is highly unlikely, as the preclinical studies indicated stability over a similar time frame. The 2 hour interval for study of Seattle-PAP was chosen, as this should allow ample time for us to assess the work of breathing on the device that reflects recruitment of the lung and differences in levels of respiratory support. The two hour periods were chosen as periods of time in which the infant will transition physiologically between the lung volume on standard Bn-CPAP and Seattle-PAP.

As stated earlier, a respiratory therapist that is an expert in all aspects of Bn-CPAP will be present throughout the 6 hour period, because we do not want the measurements of study parameters to be affected by changes arising from differences in patient management, such as with positioning of the nasal prong interfaces.

Section G: Sample Size/Data Analysis

G1. Sample Size

How many subjects (or specimens, or charts) will be used in this study?

Local: 40 Worldwide: 40

Please indicate why you chose the sample size proposed:

This will be a pilot study, and we have done a power analysis for differences in our study based on the preclinical studies done on rabbits in which studies with lavaged, sedated juvenile rabbits weighing around 1000 g were assessed for work of breathing, estimated as pressure x rate products (PRPs) comparing standard bubble nasal Bn-CPAP with Seattle-PAP, the difference in the means for PRPs in the n=12 rabbits studied was 148.24 cmH2O/min. The standard deviation of the differences, in the paired comparisons of Bn-CPAP vs. 135 degree Seattle-PAP, was 154.23. Sample size calculations for two-tailed alpha 0.05 gave n=11 and 14 for powers of 0.80 and 0.90, respectively. To allow for anticipated greater heterogeneity of the human infant population that we need to characterize, we propose to study 40 infants, with ongoing review of accumulating data by a qualified person not otherwise involved in the study.

G2. Data Analysis

Provide a description of your plan for data analysis. State the types of comparisons you plan (e.g. comparison of means, comparison of proportions, regressions, analysis of variance). Which is the PRIMARY comparison/analysis? How will the analyses proposed relate to the primary purposes of your study?

The PRIMARY comparison will be work of breathing, as estimated by pressure-rate products, assessed in each patient in each of the three periods, the first being on standard Bn-CPAP, using the Fisher-Paykel bubble CPAP device. The second study period will be with Seattle-PAP, and the third period will be with return of the patient to FP-Bn-CPAP for characterization.

The primary outcome variable is work of breathing (WOB) over a two hour period, estimated from pressure-rate products, which are assessed with 6 Fr (2 mm) catheters placed in the distal esophagus for monitoring esophageal pressures (Pes), thereby estimating changes in pleural pressures during breath cycles. The following endpoints would also be assessed: Oxygen saturations and Fraction of Inspired Oxygen (FiO2) needed to keep saturations in acceptable ranges, Heart Rates (HR), transcutaneous carbon dioxide (TcPCO2), and respiratory rates throughout the 6 hour study period. The primary outcome measure will be evaluated during the last 15 minutes of each 2 hour study period. If the subject needs clinical interventions during this period, the closest preceding 15 minutes will be used for analysis.

The pressure-rate product (PRP) data will be assessed statistically by paired t-tests, with Bonferroni corrections for multiple comparisons. Lacking any relevant human data, we estimate sample size requirements from our preclinical data, obtained in studies with juvenile rabbits. These rabbits were of one sex, relatively homogeneous genetic background, and were not born prematurely, but data estimates of differences in means and SDs of differences, with a power of 0.9, indicates 17 subjects are needed, with alpha 0.05.

Also, the study plans are to use video recording of the infants' breathing motions to test the hypothesis that methods of assessments of those recorded breathing motions can be correlated with measured PRPs to identify a method of producing objective assessments of work of breathing from video clips.

The assessments of PRPs in infants on Seattle-PAP have not been conducted previously, so we may encounter some learning curve issues, and even more likely to impact needs for additional study subjects is the even more distal efforts to characterize Work of Breathing (WOB) by analyses of video recordings. Consequently, we propose to study 40 infants.

Standard adverse events in this patient population include mortality and complications of prematurity, which include bronchopulmonary dysplasia (BPD), intraventricular hemorrhage (IVH), retinopathy of prematurity and necrotizing enterocolitis (NEC). The inclusion criteria being stable and on nasal CPAP will minimize the possibility that these complications would happen, but some infants will experience these adverse events as complications of prematurity.

Two hours of a different form of nasal CPAP is considered to be unlikely to be related to an increase in any of these adverse events. However, we will track and report these adverse events and they will be evaluated by Dr. Welty and/or Drs. Gest and Mandy. These adverse events rarely are evident in the first 3 days of a stable patient, but they will be tracked and assessed by the investigators for 28 days after the study intervention day, and 36 weeks gestational age or discharge (whichever is earlier). At a biweekly meeting of investigators, including study respiratory therapists, the patients' data will be evaluated and adverse events discussed. If in this patient population, adverse events are accumulate at a rate greater than the general population of similar patients, we would stop the study and review possible mechanisms.

Furthermore, adverse events will be reviewed according to the BCM procedures regarding potential unanticipated problems involving risks to subjects.

Section H: Potential Risks/Discomforts

H1. Potential Risks/Discomforts

Describe and assess any potential risks/discomforts and assess the likelihood and seriousness of such risks:

Standard adverse events in this patient population include mortality and complications of prematurity, which include bronchopulmonary dysplasia (BPD), intraventricular hemorrhage (IVH), retinopathy of prematurity and necrotizing enterocolitis (NEC). The inclusion criteria being stable and on nasal CPAP will minimize the possibility, but some of these infants will experience these adverse events as complications of prematurity.

Two hours of a different form of nasal CPAP is considered to be unlikely to be related to an increase in any of these adverse events. However, we will track and report these adverse events and they will be evaluated by Dr. Welty and/or Drs. Gest and Mandy. These adverse events rarely are evident in the first 3 days of a stable patient, but they will be tracked and assessed by the investigators for 28 days after the study intervention day, and 36 weeks gestational age or discharge (whichever is earlier). At a biweekly meeting of investigators, including study respiratory therapists, the patients' data will be evaluated, and adverse events discussed. If in this patient population, adverse events accumulate at rates greater than in the general population of similar patients, we would stop the study and review possible mechanisms.

While placing an esophageal catheter is not a standard procedure in our nursery placing bigger, stiffer catheters for feeding and gastric decompression is routine and is done in all patients on nasal CPAP. OG tubes are placed after delivery and have to be replaced with some frequency as needed in premature infants with rare complications. Based on the experience we have with OG tubes we regard placement of an esophageal catheter for the 6 hour duration of the study as involving minimal risk. Risks include bleeding and esophageal perforation.

We do not expect to observe any complications or safety concerns with the device, as the interface between the device and the infant will be identical to those being used currently. Standard Bn-CPAP has been shown to be an efficacious method for respiratory support and the very rare complications primarily include:

1. Breakdown of the nasal area secondary to the interface of the baby with the device at the nose. This interface is identical in the two forms of nasal CPAP so, while we will record nasal breakdown we do not anticipate any differences betweent the two devices.

2. Pneumothorax: In randomized controlled trials of Bn-CPAP versus intubation and mechanical ventilation in the delivery room the development of pneumothorax was higher in the patients randomized to nasal CPAP than those randomized to intubation and mechanical ventilation. Our rates of pneumothorax in infants under 1500 grams is around 5%, and while we do not anticipate an increase in the pneumothorax rate in treated infants we will monitor this complication.

The primary risk factors for pneumothorax in premature infants is high levels of end pressure and nasal CPAP utilization in patients with hyaline membrane disease. We will not increase CPAP in this study, and the patients will be on less than or equal to 30% oxygen indicating that respiratory system function is good, so there is no foreseeable increased risk of pneumothorax.

Dr. Welty, Dr. Mandy and Dr. Gest will be notified of the development of a pneumothorax and review whether the pneumothorax can be attributed to the device. We would not stop the study after one event as in 20 infants 1 event would be 5%. If two events were encountered the study would be stopped and the data about mechanism for pneumothorax evaluated.

Lastly, potential loss of confidentiality is a risk.

H2. Data and safety monitoring plan

Do the study activities impart greater than minimal risk to subjects?

Yes

NOTE: The answer to the questions in H2 requires the completion of the form: 'Section H – Data and Safety Monitoring Plan' as an attachment in Section S.

H3. Coordination of information among sites for multi-site research

Is the BCM Principal Investigator acting as the SPONSOR-INVESTIGATOR for this multi-site research?

No or Not Applicable

Is BCM the COORDINATING CENTER for this multi-site research?

No or Not Applicable

Section I: Potential Benefits

Describe potential benefits to be gained by the individual subject as a result of participating in the planned work.

No long term beneficial effect is expected. We think that the device will provide better support than conventional Bn-CPAP, and that the physiological endpoints assessed will document enhanced respiratory support, but we will make no provisions to continue the experimental device as conventional Bn-CPAP is presently the TCH nursery standard for clinical care.

Describe potential benefits to society of the planned work.

These pilot data are essential and if the results are favorable a non-inferiority trial and potential efficacy trial would follow. The goal is to improve non-invasive respiratory support, limiting acute lung injury and diminish chronic respiratory morbidity in a population highly susceptible to these complications of prematurity and this pilot study is essential to do to determine whether this device is reasonable in this sequence of studies with the long term potential beneficial effect to reduce the incidence of BPD and/or chronic respiratory morbidity.

Do anticipated benefits outweigh potential risks? Discuss the risk-to-benefit ratio.

Bn-CPAP supports spontaneous breathing by delivering a continuous, pressurized gas flow to an infant's airway. The gas is usually humidified air, enriched with oxygen, and is delivered to the infant's nose though a breathing circuit and nasal prongs. The pressure of the delivered gas is controlled by simply adjusting the depth of a partially submerged tube attached to the end of the infant's breathing circuit. Bn-CPAP may provide additional benefits over conventional nasal CPAP systems because as gas exits the submerged tube it forms bubbles that create small airway pressure oscillations. These oscillations are transmitted to the patient's lungs and are thought to improve gas exchange, enhance lung recruitment and reduce the work of breathing.

Seattle-PAP device improves upon conventional Bn-CPAP by providing an equivalent mean airway pressure accompanied by oscillations that may reduce the likelihood an infant will need to receive mechanical ventilation. The key difference of the Sea-PAP device is that the expiratory tube is placed in the water at a 135° angle; conventional Bn-CPAP tubes are placed in the water at 0° (straight down). It is this change in angle that more consistently produces the range of oscillations thought to improve lung function and make it easier for an infant to breathe.

The risk to benefit ratio is favorable.

Section J: Consent Procedures

J1. Waiver of Consent

Will this research require a waiver of consent and authorization?

No

Will additional pertinent information be provided to subjects after participation?

No

Explain why providing subjects additional pertinent information after participation is not appropriate.

J1a. Waiver of requirement for written documentation of Consent

Is this research subject to FDA regulations?

No

Explain how the research involves no more than minimal risk to the participants, and the specifics demonstrating that the research does not involve procedures for which written consent is normally required outside of the research context.

Explain how the only record linking the participant and the research would be the consent document, and how the principal risk would be potential harm resulting from a breach of confidentiality, and how each participant will be asked whether he or she wants documentation linking the participant with the research and their wishes will govern.

J2. Consent Procedures

Who will recruit subjects for this study?

PI
PI's staff

Describe how research population will be identified, recruitment procedures, and consent procedures in detail.

Subjects admitted to TCH or Pavilion for Women (PFW) will be identified by the study physicians for potential enrollment into the study. The new admissions and Labor & Delivery EPIC lists will be scanned every day to identify potential subjects. Admit date, gestational age and admitting diagnosis will be included in the lists. All subjects with Bn-CPAP will be recruited and enrolled if they meet the eligibility criteria. Once identified, study physicians will approach the parent or guardian for consent.

The subject's choice whether or not to participate will in no way affect the present or future care they receive from their physician. Written informed consent will be obtained from the parent/legal guardian according to federal regulations and BCM policies by the PI, co-I and/or study staff.

If needed, the short-form Spanish consent document will be used to consent Spanish speaking patients, in conjunction with a Spanish interpretation of full length English consent. The short-form Spanish consent document is attached in Section S. A certified institutional translator (or bi-lingual study staff) will help facilitate the consent discussion. A non-biased witness will be present during the consent discussion.

Are foreign language consent forms required for this protocol?

Yes

Which of the following ways will you document informed consent in languages other than English?

Short-Form consent documents

J3. Privacy and Intrusiveness

Will the research involve observation or intrusion in situations where the subjects would normally have an expectation of privacy?

No

J4. Children

Will children be enrolled in the research?

Yes

J5. Neonates

Will non-viable neonates or neonates of uncertain viability be involved in research?

No

J6. Consent Capacity - Adults who lack capacity

Will Adult subjects who lack the capacity to give informed consent be enrolled in the research?

No

J7. Prisoners

Will Prisoners be enrolled in the research?

No

Section K: Confidentiality

Will research data include health information by which subjects can be identified?

Yes

Where will research data be kept? How will such data be secured?

An institutional Electronic Data Capture (EDC) system, the Clinical Trials Management System (CTMS), will be used by BCM and TCH for this trial. Study-specific electronic case report forms (eCRFs) designed to provide reliable and secure data entry for clinical research purposes will be utilized. The web-based software tools in the CTMS employ 2048-bit Secure Socket Layer (SSL) encryption; all transactions are encrypted in both directions. The CTMS meets the regulatory requirements of HIPAA and 21 CFR Part 11 compliance. In addition, the system has an offsite, fully mirrored disaster recovery backup.

All data changes in the CTMS are written to an audit trail that identifies the user, date and time, as well as the old value and new value. Both patient-related data and trial configuration data are written to the audit trail. Data are saved at regular intervals during data entry, to prevent loss of information in the event of a disruption of the Internet connection.

The eCRFs include standard logical checks and range checks, as well as support for multiple languages in the user interface. There is emphasis on tracking deadlines and milestones with automated notifications and reports of study progress. The module includes a data locking mechanism, to ensure that data accuracy and integrity are maintained to facilitate data analyses promptly after study completion.

Several levels of security are employed to ensure privacy and integrity of the study data, including the following:

• Study access requires use of assigned unique user names and passwords that are modified at specified time intervals. • Individual roles and access levels are assigned. • Passwords are changed regularly. • Data are not stored on laptop computers. The study coordinator will be responsible for data entry and maintaining appropriate source documentation. Source documentation refers to original records of observations, clinical findings, and evaluations that are subsequently recorded as data.

Paper Research Records:

Very little data will be obtained and/or stored on paper. The limited data on paper will be stored in notebooks specific to each enrollee. The notebooks will be stored in a locked file cabinet with space designated for the study only. The file cabinet will be in research space which is locked and unavailable to the public.

Who, besides the PI, the study staff, the IRB and the sponsor, will have access to identifiable research data?

No one else will have access.

Will you obtain a Certificate of Confidentiality for this study?

No

Please further discuss any potential confidentiality issues related to this study.

Section L: Cost/Payment

Delineate clinical procedures from research procedures. Will subject's insurance (or subject) be responsible for research related costs? If so state for which items subject's insurance (or subject) will be responsible (surgery, device, drugs, etc). If appropriate, discuss the availability of financial counseling.

The subject will not be responsible for research related costs.

If subjects will be paid (money, gift certificates, coupons, etc.) to participate in this research project, please note the total dollar amount (or dollar value amount) and distribution plan (one payment, pro-rated payment, paid upon completion, etc) of the payment.

Dollar Amount:
0

Distribution Plan:

Section M: Genetics

How would you classify your genetic study?

Discuss the potential for psychological, social, and/or physical harm subsequent to participation in this research. Please discuss, considering the following areas: risks to privacy, confidentiality, insurability, employability, immigration status, paternity status, educational opportunities, or social stigma.

Will subjects be offered any type of genetic education or counseling, and if so, who will provide the education or counseling and under what conditions will it be provided? If there is the possibility that a family's pedigree will be presented or published, please describe how you will protect family member's confidentiality?

Section N: Sample Collection

None

Section O: Drug Studies

Does the research involve the use of ANY drug* or biologic? (*A drug is defined as any substance that is used to elicit a pharmacologic or physiologic response whether it is for treatment or diagnostic purposes)
No

Does the research involve the use of ANY gene transfer agent for human gene transfer research?
No

O1. Current Drugs

Is this study placebo-controlled?
No

Will the research involve a radioactive drug that is not approved by the FDA?
No

Section P: Device Studies

Does this research study involve the use of ANY device?
Yes

Device 1: Seattle-CPAP

Section Q: Consent Form(s)

Informed Consent

Section R: Advertisements

None
