# Supplementary material for: Short term evaluation of respiratory effort by premature infants supported with bubble nasal continuous airway pressure using Seattle-PAP and a standard bubble device
Source: PLoS One. 2018 Mar 28;13(3):e0193807. doi: 10.1371/journal.pone.0193807 (PMC5874011; doi:10.1371/journal.pone.0193807)
Supplement: S3 Text — This is a copy of the consent form for the study, as approved by the Baylor/TCH IRB. (PDF) [file pone.0193807.s007.pdf]

H-29620- A STUDY TO EVALUATE THE EFFICACY OF SEATTLE-PAP FOR THE RESPIRATORY SUPPORT OF PREMATURE INFANTS

Background

This Informed consent form gives you important information about this study that will be described in detail. We will answer all questions you may have and explain any words or information in this consent form you do not clearly understand. You should understand the information in this document before volunteering to have your baby participate in this study.

Patients that are born eight weeks or more too early almost always have some problems with their lungs because they are premature. They need to be treated with good care to make their lungs work better so that oxygen can get into the blood and carbon dioxide, a waste product, can be eliminated. Sometimes premature babies need to be placed on a respirator (breathing machine) which helps the lungs work better. But, the respirator may also damage the lungs.

The safest way to help the lungs work better is with something called Nasal CPAP. CPAP stands for continuous positive airway pressure and means that pressure is applied to the lungs to keep them full of air so that they can absorb oxygen and eliminate carbon dioxide.

The way Nasal CPAP works is that we turn on a gas flow (containing oxygen and air) and attach it with a piece of plastic to the baby's nose (nasal prong). The nasal prong is attached to a tube that flows to a bottle of water. The amount of pressure in the tubing is equal to how far the tubing is put into water. Similar types of devices are used frequently in adults who have trouble breathing when they sleep (apnea).

The water bubbles as the gas that goes into the water comes out of the other end of the tubing. So, we call this kind of Nasal CPAP - Bubble Nasal CPAP (Bn-CPAP). There is research that suggests the bubbles actually help the baby, too. In other words, both the pressure and the bubbling may help the babies' lungs stay filled with air.

When Bn-CPAP is used, the babies still need to do their own breathing. Therefore, about half the time babies on Bn-CPAP need to be placed on a respirator because they forget to breathe or their lungs are too sick.

It would help many babies across the world to find a better way to do nasal CPAP and that is what we are trying to do with this research study. We have done some studies on small animals which showed us that a different kind of nasal CPAP, which we call Seattle-PAP works better than Bn-CPAP.

This research study is sponsored by Seattle Children’s Hospital Foundation. Seattle Children’s Hospital Foundation receives funding from Bill & Melinda Gates Foundation to do the study.

Purpose

In this study, we will place premature babies that are on Bn-CPAP on Seattle-PAP for 2 hours. We will take measurements when the baby is on Bn-CPAP and Seattle-PAP, respectively. If Seattle-PAP works better, we will see that the babies do not breathe as fast or as hard on the new device, and they will not need as much oxygen as with Bn-CPAP.

What we learn from this research study will help us determine whether we should do a larger study to compare the effectiveness of the two devices.

Procedures
The research will be conducted at the following location(s): Baylor College of Medicine, Seattle Children's Hospital - Washington, TCH: Texas Children's Hospital.

This is a single site study and we plan to enroll approximately 40 babies at our site.

If you agree to have your baby to take part in this study, we will obtain informed consent and determine if your baby is eligible to take part in this study. Babies that are between 6 and 72 hours old and are doing well on Bn-CPAP will be included in the study.

1. Nasal CPAP:

During this study we will use two types of nasal CPAP: Bn-CPAP and Seattle-PAP

Your baby will be first placed on Bn-CPAP (~120 minutes), then switched to Seattle-PAP (~120 minutes), and finally switched back to Bn-CPAP (~ 120 minutes). Your baby's participation in the study will end after this 6 hour period. We will use the Bn-CPAP and Seattle-PAP machines provided by the sponsor during the study period.

After we are done with the study procedures, your baby will be switched back to the Bn-CPAP machine routinely used in our nursery.

We do not think that your baby will get worse during the two hours that they are on Seattle-PAP because all the studies that we have done so far show that the Seattle-PAP works better than Bn-CPAP. But, if your baby gets worse, we would change back to Bn-CPAP sooner.

The study team will follow your baby's condition for safety reasons up to 28 days from end of study AND 36 weeks gestational age OR discharge (whichever occurs first).

2. Lung Pressure Monitoring:

A small tube will be placed in the esophagus (food pipe), which connects the mouth to the stomach, to measure pressure in your baby's chest. This is a good and safe way to measure the pressure. As the baby breathes, the amount of pressure that the baby needs to breathe will be measured. When babies breathe hard, the pressure that is measured is high. This tube will be connected to a special machine called "DARCI" (DAta ReCording Instrument), which will continuously record chest pressure data.

3. Carbon dioxide Monitoring:

Your baby's carbon dioxide levels will also be continuously monitored during the study period. A ring will be securely attached to your baby's skin to connect him/her to the monitoring device. The

adhesive used to attach the ring may cause some skin irritation.

4. Video Monitoring:

During the study periods on both types of nasal CPAP, we may obtain videos of your baby's breathing. We will do this to compare your baby's chest and belly movements to the chest pressure readings.

We will protect your baby's identity by placing shields over your baby's eyes. These eye shields are used in many babies that need light for jaundice, and there is no risk associated with using the shields.

5. We will collect your baby's demographic (age, race, ethnicity) and labor/delivery information.

6. We will also record results from any other monitoring tools that your baby's doctor is using as part of his/her routine care. We will collect this data continuously for the 6 hour study period. This will help us identify patterns that might lead to issues in premature babies. We will also be studying the effects of any medications your baby's doctor gives your baby.

Once enrolled in the study, your baby will be given a unique identification (ID) number (coded). His/her personal information (for example name, date of birth, address) will not be attached to the data collected for this study. The link between the unique ID number and your baby's personal information will be kept in a separate file. The access to this file will be limited to your study doctor and study staff.

You can see and get a copy of your research related health information. Your research doctor may be able to provide you with part of your information while the study is in progress and the rest of your information at the end of the study.

Potential Risks and Discomforts
Since the babies joining this study are premature, they may experience the following complications:

- Bronchopulmonary Dysplasia (Chronic lung condition that affects newborn babies who were either put on a respirator or not. This is a chronic condition that is usually long-lasting and does not easily or quickly go away.),
- Intraventricular Hemorrhage (bleeding in the brain),
- Retinopathy of Prematurity (eye problems),
- Necrotizing Enterocolitis (gut becomes infected and can begin to die),
- Pneumothorax (air leak in lungs),
- Death.

We will exclude subjects that are not already stable on standard Bn-CPAP, to reduce the possibility, but some of the babies may still experience above complications. It is unlikely that two hours of a different form of nasal CPAP could be related to an increase in any of these complications.

While placing an esophageal catheter (tube in your food pipe) for measuring pressures is not a standard procedure in our nursery, placing bigger, stiffer tubes for routine care is common and is done in all patients on standard nasal CPAP. These tubes will be placed after delivery and have to be replaced with some frequency, as needed in premature infants, and complications with these procedures are rare. Based on our experience, we regard placement of an esophageal catheter for the 6 hour duration of the study as involving minimal risk. Risks include bleeding and esophageal perforation (hole in the food pipe).

We do not expect to observe any complications or safety concerns with Seattle-PAP, as the interface between the device and the infant will be identical to those being used currently. Standard Bn-CPAP has been shown to be an effective method for respiratory support, and the very rare complications primarily include:

- Breakdown of the skin in the nasal area. Seattle-PAP is attached to the babies' noses in the same manner as the standard Bn-CPAP. Therefore, we do not believe that Seattle-PAP will place your baby at additional risk.

- About 5% of babies on standard Bn-CPAP develop pneumothorax, which is when the lung collapses and air fills up the chest but is on the outside of the lung. This is a serious problem and the baby can get sick because of it. We have no reason to think that getting a pneumothorax is more likely to happen when using Seattle-CPAP, but we will make certain of that in this study. If two babies develop a pneumothorax, the study would be stopped

Study staff will update you in a timely way on any new information that may affect your decision to stay in the study.

Potential Benefits
The benefits of participating in this study may be: that your baby does not breathe as fast or as hard on Seattle-PAP, and that your baby may not need as much oxygen as with Bn-CPAP. Also, what we learn from this research study will help us determine whether we should do a larger study to compare the effectiveness of the two devices. However, you may receive no benefit from participating.

Alternatives
The following alternative procedures or treatments are available if you choose not to participate in this study: your baby will receive treatment for your his/her lung condition that your medical team thinks is best and your baby may get treated with standard nasal CPAP; which is our nursery standard.

Subject Withdrawal from a Study

You can decide to stop having your baby take part in this study at any time. Tell the study doctor if you are thinking about stopping or decide to stop. He or she will tell you how to stop safely. The study doctor will also discuss what follow-up care and testing would be most helpful for your baby. The data collected before you stop the study may still be used by your study doctor.

Investigator Withdrawal of Subject from a Study

The investigator or sponsor may decide to stop you from taking part in this study at any time. You could be removed from the study for reasons related only to you (for example, if you move to another city, or if you have a serious reaction to your study treatment) or because the entire study is stopped. The sponsor, investigator, Food and Drug Administration, or Institutional Review Board may stop the study at any time.

Subject Costs and Payments

You will not be asked to pay any costs related to this research.

You will not be paid for taking part in this study.

Research Related Injury

Immediate necessary medical care is available at Texas Children's Hospital/Baylor College of Medicine (TCH/BCM) if your baby is injured as a result of being in this research study.

Research personnel will try to reduce, control, and treat any complications from this research. If your baby is injured because of this study, the sponsor will pay for any reasonable medical treatment costs incurred by the patient who is harmed as a result of participation.

Subject's Rights

Your signature on this consent form means that you have received the information about this study and that you agree to volunteer for this research study.

You will be given a copy of this signed form to keep. You are not giving up any of your rights by signing this form. Even after you have signed this form, you may change your mind at any time. Please contact the study staff if you decide to stop taking part in this study.

If you choose not to take part in the research or if you decide to stop taking part later, your benefits and services will stay the same as before this study was discussed with you. You will not lose these benefits, services, or rights.

Your Health Information

We may be collecting health information that could be linked to you (protected health information). This protected health information might have your name, address, social security number or something else that identifies you attached to it. Federal law wants us to get your permission to use your protected health information for this study. Your signature on this form means that you give us permission to use your protected health information for this research study.

If you decide to take part in the study, your protected health information will not be given out except as allowed by law or as described in this form. Everyone working with your protected health information will work to keep this information private. The results of the data from the study may be

published. However, you will not be identified by name.

People who give medical care and ensure quality from the institutions where the research is being done, the sponsor(s) listed in the sections above, representatives of the sponsor, agents of the Food and Drug Administration, and regulatory agencies such as the U.S. Department of Health and Human Services will be allowed to look at sections of your medical and research records related to this study. Because of the need for the investigator and study staff to release information to these parties, complete privacy cannot be guaranteed.

The people listed above will be able to access your information for as long as they need to, even after the study is completed.

If you decide to stop taking part in the study or if you are removed from the study, you may decide that you no longer allow protected health information that identifies you to be used in this research study. Contact the study staff to tell them of this decision, and they will give you an address so that you can inform the investigator in writing. The investigator will honor your decision unless not being able to use your identifiable health information would affect the safety or quality of the research study.

The investigator, STEPHEN WELTY, and/or someone he/she appoints in his/her place will try to answer all of your questions. If you have questions or concerns at any time, or if you need to report an injury related to the research, you may speak with a member of the study staff: DR. STEPHEN WELTY at 832-826-1380 during the day and the doctor on call at night at 832-826-4385.

Members of the Institutional Review Board for Baylor College of Medicine and Affiliated Hospitals (IRB) can also answer your questions and concerns about your rights as a research subject. The IRB office number is (713) 798-6970. Call the IRB office if you would like to speak to a person independent of the investigator and research staff for complaints about the research, if you cannot reach the research staff, or if you wish to talk to someone other than the research staff.

If you sign this form and your child participates in the study, the study staff will be authorized to use the information described above to carry out the purposes of the research study. The study staff will also be authorized to disclose the information described above to the following parties involved in the research study:

• Seattle Children's Research Institute, SCRI (Sponsor), Intellectual Ventures Labs, IV Lab, or other legal agents designated by sponsor to collect or review study data.
• The Institutional Review Board (IRB) or Independent Ethics Committee (IEC) that oversees the research study at your site.
• Government agencies including the FDA.

A description of this clinical trial will be available on http://www.ClinicalTrials.gov as required by U.S. Law. This Web site will not include information that can identify you. At most, the Web site will include a summary of the results. You can search this Web site at any time. Federal law requires clinical trial information for certain clinical trials to be submitted to the data bank.

CONSENT FORM

Institutional Review Board for Baylor College of Medicine and Affiliated Hospitals

Informed Consent

H-29620- A STUDY TO EVALUATE THE EFFICACY OF SEATTLE-PAP FOR THE RESPIRATORY SUPPORT OF PREMATURE INFANTS

Signing this consent form indicates that you have read this consent form (or have had it read to you), that your questions have been answered to your satisfaction, and that you voluntarily agree to participate in this research study. You will receive a copy of this signed consent form.

______________________________________ ______________

Subject Date

______________________________________ ______________

Legally Authorized Representative Date
Parent or Guardian

______________________________________ ______________

Investigator or Designee Obtaining Consent Date

______________________________________ ______________

Witness (if applicable) Date

______________________________________ ______________

Translator (if applicable) Date
