# Supplementary material for: Short term evaluation of respiratory effort by premature infants supported with bubble nasal continuous airway pressure using Seattle-PAP and a standard bubble device
Source: PLoS One. 2018 Mar 28;13(3):e0193807. doi: 10.1371/journal.pone.0193807 (PMC5874011; doi:10.1371/journal.pone.0193807)
Supplement: S2 Text — This is a copy of the letter of approval issued by the Baylor/TCM IRB. (PDF) [file pone.0193807.s006.PDF]

June 27, 2014

Baylor College of Medicine
Office of Research
One Baylor Plaza, 600D
Houston, Texas 77030
Phone: (713) 798-6970
Fax: (713) 798-6990
Email: irb@bcm.tmc.edu

STEPHEN WELTY
BAYLOR COLLEGE OF MEDICINE
PEDIATRICS: NEWBORN

**H-29620 - A STUDY TO EVALUATE THE EFFICACY OF SEATTLE-PAP FOR THE RESPIRATORY SUPPORT OF PREMATURE INFANTS**

**APPROVAL VALID FROM 2/6/2014 TO 2/5/2015**

Dear Dr. WELTY

The Institutional Review Board for Human Subject Research for Baylor College of Medicine and Affiliated Hospitals (BCM IRB) is pleased to inform you that the research protocol and consent form(s) named above were approved.

The study may not continue after the approval period without additional IRB review and approval for continuation. You will receive an email renewal reminder notice prior to study expiration; however, it is your responsibility to assure that this study is not conducted beyond the expiration date.

Please be aware that only IRB-approved informed consent forms may be used when written informed consent is required.

Any changes in study or informed consent procedure must receive review and approval prior to implementation unless the change is necessary for the safety of subjects. In addition, you must inform the IRB of adverse events encountered during the study or of any new and significant information that may impact a research participants' safety or willingness to continue in your study.

The BCM IRB is organized, operates, and is registered with the United States Office for Human Research Protections according to the regulations codified in the United States Code of Federal Regulations at 45 CFR 46 and 21 CFR 56. The BCM IRB operates under the BCM Federal Wide Assurance No. 00000286, as well as those of hospitals and institutions affiliated with the College.

Sincerely yours,

VERNON R SUTTON, M.D., B.S.
Institutional Review Board for Baylor College of Medicine and Affiliated Hospitals
